# Non-canonical signaling hubs upon inhibition of MyD88-ERK interaction in cancer

**DOI:** 10.1038/s41419-026-09131-z

**Published:** 2026-07-30

**Authors:** Nader Hussein, Najwa Skafi, Thuy Ha Pham, Melina Gautier, Francois Virard, Frédérique Fauvet, Léa Magadoux, Christelle Chassot-Lamblot, Anne-Pierre Morel, Christophe Vanbelle, Bassam Badran, Serge Lebecque, Serge Nicolas Manié, Sylvain Lamure, Toufic Renno, Isabelle Coste

**Affiliations:** 1 Centre de Recherche en Cancérologie de Lyon, Université Claude Bernard Lyon 1, INSERM 1052, CNRS UMR 5286, Centre Léon Bérard, Lyon, France; 2 Laboratory of Cancer Biology and Molecular Immunology, Department of Chemistry and Biochemistry, Faculty of Science I, Lebanese University, Hadat, Lebanon; 3 Faculté d’Odontologie, Hospices Civils de Lyon, University of Lyon, Lyon, France; 4 Faculté de Médecine, Hospices Civils de Lyon, University of Lyon, Lyon, France; 5 Hematology Department-Centre Léon Bérard, Lyon, France

**Keywords:** Cancer, Cell biology

## Abstract

The RAS-MAPK pathway is frequently dysregulated in cancer, leading to malignant transformation. We previously identified a direct interaction between MyD88, a central adaptor of inflammatory signaling, and ERK, the terminal kinase of the RAS-MAPK cascade. Pharmacologic disruption of this ERK-MyD88 complex with a small molecule induces immunogenic cell death and antitumor immunity, suggesting a novel therapeutic strategy. Here, we show that ERK-MyD88 inhibition drives cellular signaling from non-canonical hubs. First, ERK-MyD88 disruption activates an integrated stress response that drives caspase-8-dependent apoptosis through a FADD-independent pathway within p62-ubiquitin aggregates. Second, it promotes assembly of MyD88, IRAK1, and IRAK4 into Myddosomes that produce proinflammatory chemokines independently of TLR or IL-1R activation. These findings demonstrate that perturbation of the ERK-MyD88 complex rewires cellular signaling into cell-autonomous hubs that coordinate caspase activation with chemokine secretion, thereby driving a program of immunogenic cancer cell death.

## Introduction

The RAS-RAF-MEK-ERK signaling pathway plays a fundamental role in the control of key cellular processes, including cell survival and proliferation [1]. This pathway is hyperactivated in a high percentage of tumors, with between 20 and 30 percent of human cancers harboring activating mutations of the KRAS, NRAS, and BRAF genes [2,3].

We previously reported that ERK, the distal kinase of this pathway, interacts with MyD88, an adaptor protein of the TLR/IL1-R inflammatory pathway. This interaction sustains ERK signaling, linking RAS oncogenic signaling with inflammation, transformation, and cancer cell survival [4,5]. More recently, we identified inhibitors of the ERK-MyD88 interaction, including the proof-of-concept compound EI-52, which induces early immunogenic apoptotic cell death and stimulates an anti-tumor T-cell response in vivo, thereby highlighting a novel therapeutic strategy for cancer treatment [6].

While our earlier work established that disrupting this interaction activates an integrated stress response (ISR) and immunogenic cell death, the underlying mechanism involved remained to be elucidated. Here, we show that disruption of the ERK-MyD88 complex results in the emergence of non-canonical signaling hubs that arise concurrently with p62-positive ubiquitin aggregates, driving caspase-8 activation via a non-canonical, FADD-independent pathway. Furthermore, we show that ERK-MyD88 interaction inhibition leads to the assembly of active, receptor-independent Myddosomes, which mediate chemokine production independent of canonical Toll-like receptor (TLR) or IL-1 receptor activation.

## Results

### ERK-MyD88 interaction blockade induces caspase-8 activation through a FADD-independent, non-canonical pathway

**Fig. 1:**
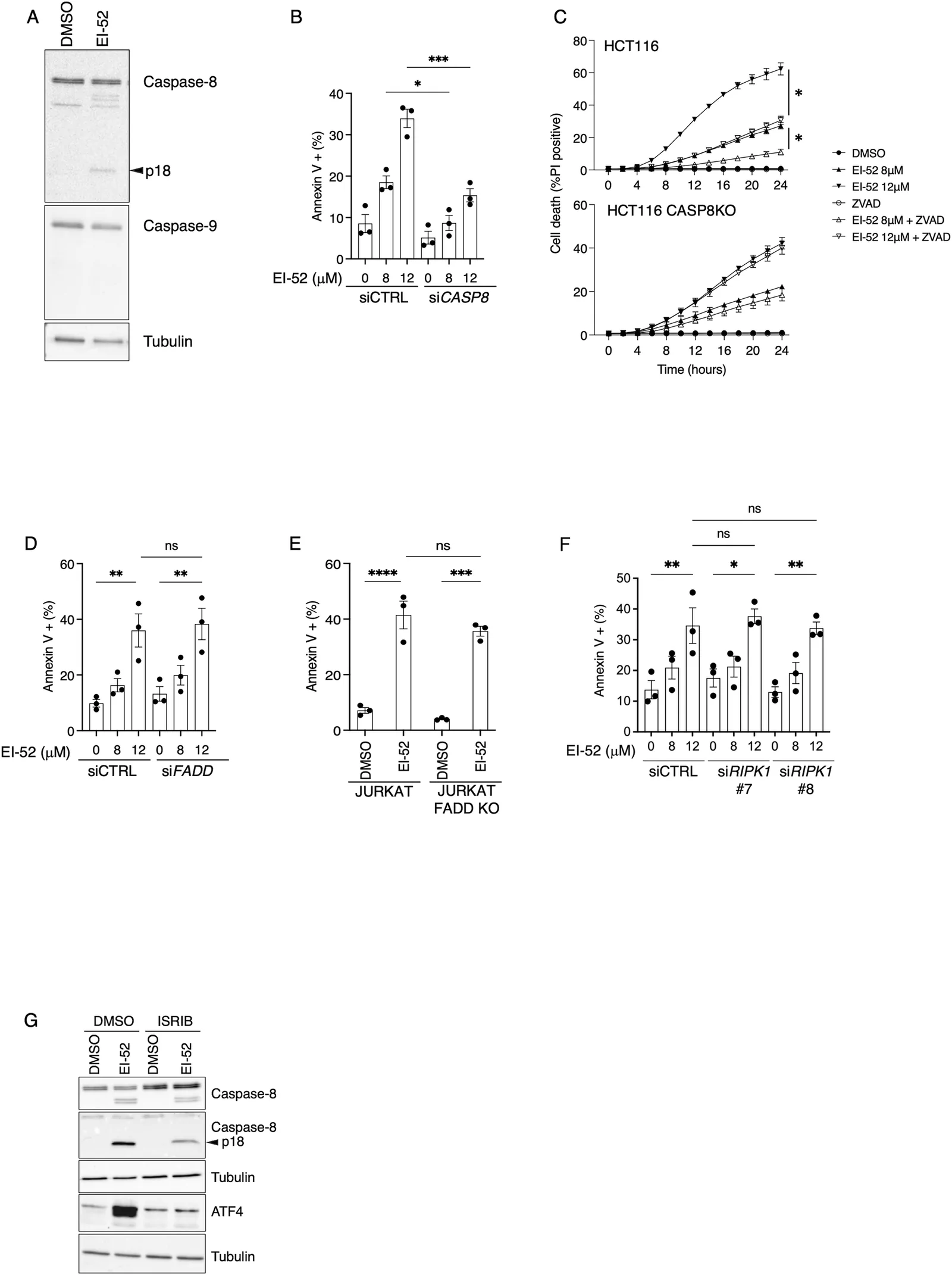
Non-canonical Caspase-8 activation occurs independently of FADD following ERK-MyD88 interaction inhibition. **A** HCT116 cells were treated for 8 h with DMSO or EI-52 8 μM. Caspase 8 and 9 cleavage was evaluated by western blot. Representative data of three independent experiments. **B** HCT116 cells were transfected with *Caspase 8* or Control siRNA for twenty-four hours. Then, the cells were treated with DMSO or 8 or 12 μM of EI-52 for 8 h. Apoptosis (annexin V/PI) was analyzed by flow cytometry. Mean percentage of annexin V^+^ cells ± SEM from three independent experiments, Tukey’s one-way ANOVA, EI-52 8 μM siCTRL vs si*CASP8* *p = 0.0242, EI-52 12 μM siCTRL vs siC*ASP8* ***p = 0.0001 (95% confidence interval). **C** HCT116 WT or caspase 8-deficient was treated with DMSO or 8 or 12 μM of EI-52 with or without 25 μM of ZVAD, a pan-caspase inhibitor, for 24 h. Cell death was evaluated by measuring the percentage of propidium iodide-positive cells over time. Results are expressed as mean ± SEM from three independent experiments. Statistical analysis was performed using two-way ANOVA; *p < 0.05. **D** HCT116 cells were transfected with *FADD* or Control siRNA for twenty-four hours. Then, the cells were treated with DMSO or 8 or 12 μM of EI-52 for eight hours. Apoptosis (annexin V/PI) was analyzed by flow cytometry. Mean percentage of annexin V+ cells ± SEM from three independent experiments, Tukey’s one-way ANOVA siCTRL DMSO vs EI-52 12 μM **p = 0.0056, si*FADD* DMSO vs EI-52 12 μM **p = 0.0029, EI-52 12 μM siCTRL vs si*FADD* ns *p* = 0.9980. **E** JURKAT and JURKAT FADD-KO cells were treated with EI-52 8 μM for eight hours. Apoptosis (annexin V/PI) was analyzed by flow cytometry. Mean percentage of annexin V^+^ cells ± SEM from three independent experiments, Tukey’s one-way ANOVA DMSO vs EI-52 WT ****p < 0.0001, DMSO vs EI-52 FADD-KO ***p = 0.0002, EI-52 WT vs EI-52 FADD-KO ns *p* = 0.4644. **F** HCT116 cells were transfected with *RIPK1#7 or #8* or Control siRNA for 24 h. Then, the cells were treated with DMSO or 8 or 12 μM of EI-52 for eight hours. Apoptosis (annexin V/PI) was analyzed by flow cytometry (upper panel). Mean percentage of annexin V^+^ cells ± SEM from three independent experiments, Tukey’s one-way ANOVA, siCTRL DMSO vs EI-52 12 μM **p = 0.0080, si*RIPK1#7* DMSO vs EI-52 12 μM *p = 0.0111, si*RIPK1#8* DMSO vs EI-52 12 μM **p = 0.0082 EI-52 12 μM siCTRL vs si*RIPK1#7* ns *p* = 0.9989, EI-52 12 μM siCTRL vs si*RIPK1#8* ns *p* > 0.9999 (95% confidence interval). **G** HCT116 cells were pre-incubated or not with ISRIB 400 nM for 1 h, then treated with EI-52 8 μM for 16 h. Caspase 8 cleavage, ATF4 and tubulin were evaluated by western blot. Representative data of three independent experiments.

To further characterize the cancer cell apoptosis triggered by the disruption of ERK-MyD88 interaction, we evaluated the activation of initiator caspases. In HCT116 cells treated with EI-52 for 8 h, caspase-8, but not caspase-9, was cleaved (Fig. 1A), indicating activation of the extrinsic rather than the intrinsic apoptotic pathway. Moreover, caspase-8 activation upon ERK-MyD88 interaction inhibition does not result from the secondary, mitochondrial route, since the latter requires caspase-9, which is not activated following treatment by EI-52, even at later time points, such as 16 h (Supplementary Fig. 1A). In addition, the percentage of Annexin V-positive apoptotic cells was reduced by fifty percent in caspase-8-silenced cells after EI-52 treatment, demonstrating the involvement of caspase-8 in EI-52-induced apoptosis (Fig. 1B and Supplementary Fig. 1B). To determine whether the residual cell death results from incomplete caspase-8 extinction or from a caspase-independent mechanism, we assessed cell death in parental HCT116 (HCT116_SCR) and in HCT116 deficient for caspase 8 (HCT116_CASP8KO) treated with EI-52 in presence or absence of the pan-caspase inhibitor ZVAD. EI-52-induced cell death was reduced by ZVAD in HCT116_SCR cells, whereas ZVAD had no additional effect in caspase-8 deficient cells (Fig. 1C and Supplementary Fig. 1C). Collectively, these results indicate that caspase 8 is the only initiator caspase activated following EI-52 treatment.

Mechanistically, caspase-8 is known to be activated by extrinsic signals emanating from death receptors, a process that requires an oligomerization step involving the recruitment of its DED domain by the adaptor molecule FADD [7,8]. Surprisingly, silencing of FADD expression with a specific siRNA had no effect on EI-52-induced apoptosis, suggesting that FADD is not required for caspase 8-dependent apoptosis in this context (Fig. 1D and Supplementary Fig. 1D). To rule out potential effects of residual FADD remaining after siRNA-mediated knockdown, we compared Annexin V externalization in FADD WT and KO cells. Following EI-52 treatment, the percentage of Annexin V^+^ cells was similar between cells with or without FADD expression (Fig. 1E and Supplementary Fig. 1E). Moreover, silencing of RIPK1, another DISC component [9], also failed to reduce cell death levels induced by EI-52 treatment (Fig. 1F and Supplementary Fig. 1F). These results reveal a non-canonical mechanism for the activation of caspase-8 upon inhibition of ERK-MyD88 interaction.

The finding that apoptosis accounts for half the observed cell death triggered by EI-52 suggests the presence of a complementary caspase-independent cell death mechanism. Given the established interplay between caspase-8 signaling and necroptosis [10], we evaluated whether EI-52-induced caspase-independent cell death involved activation of the necroptotic pathway. Treatment with necrosulfamide, a necroptosis inhibitor, did not rescue cell viability in either condition (Supplementary Fig. 1G), indicating that necroptosis is unlikely to contribute to the observed cell death.

### Aggregation-driven caspase-8 activation as a consequence of ERK-MyD88 disruption

We previously demonstrated that the integrated stress response (ISR) contributes to EI-52-induced apoptosis [6]. To investigate the role of the ISR in caspase-8 cleavage, we pretreated HCT116 cells with ISRIB, an inhibitor of phosphorylated-eIF2α, following EI-52 treatment. Caspase-8 cleavage, like ATF4 expression, was markedly reduced in ISRIB-pretreated cells (Fig. 1G), thereby confirming the involvement of the ISR in caspase-8-dependent, FADD-independent apoptosis.

**Fig. 2:**
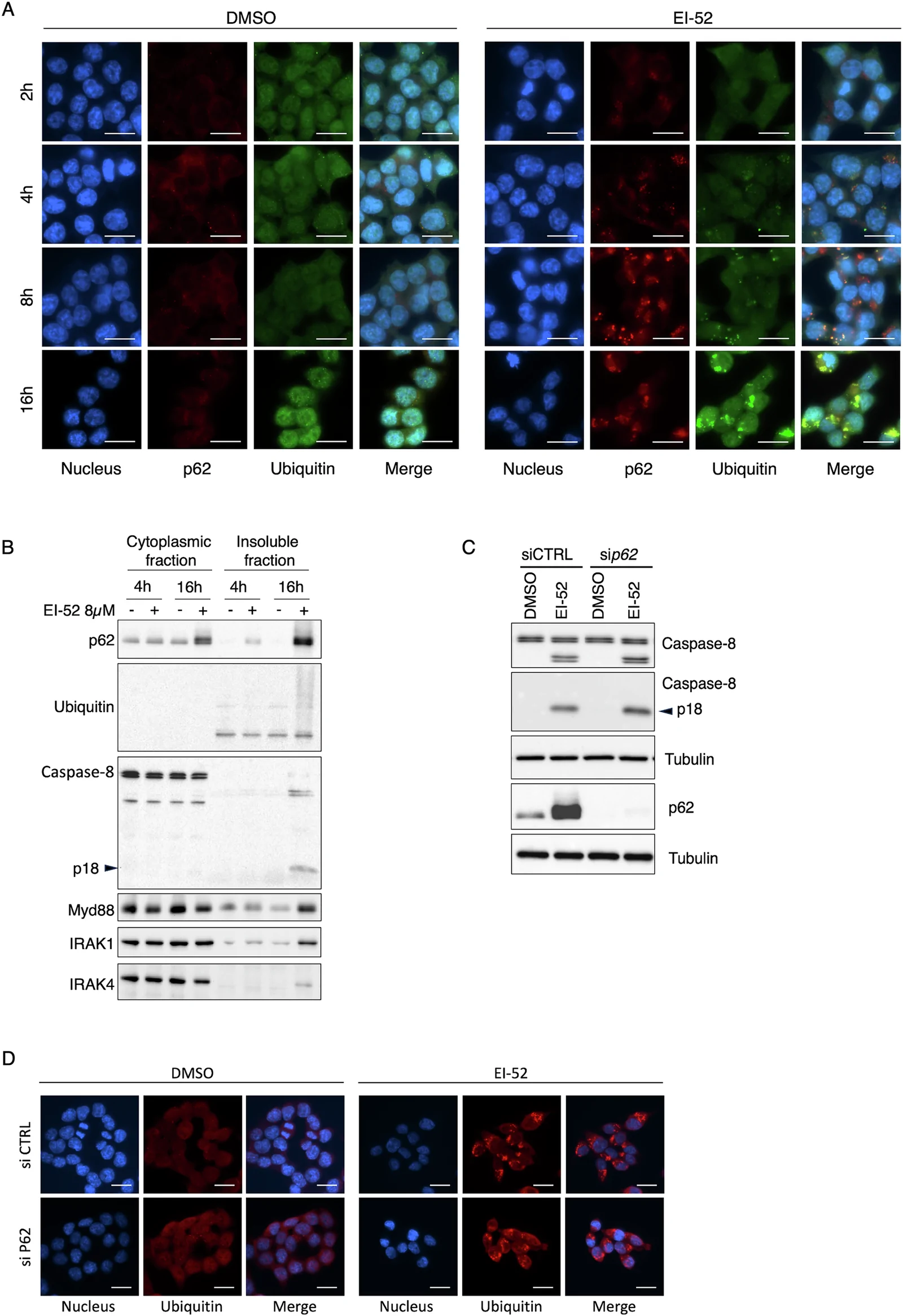
ERK-MyD88 interaction inhibition induces protein aggregate formation. **A** Immunofluorescence staining of p62 and ubiquitin. HCT116 cells were treated for 2, 4, 8, and 16 h with DMSO or EI-52 8 μM. The bar represents 20 μm. Representative data of three independent experiments. **B** HCT116 cells were treated for 4 or 16 h with DMSO or EI-52 8 μM. Proteins were extracted from cytosolic and insoluble fractions and p62, Ubiquitin, Caspase-8, MyD88, IRAK1 and IRAK4 were analyzed by western blot. Representative data of three independent experiments. **C** HCT116 cells were transfected with *p62* or Control siRNA for twenty-four hours. Then, the cells were treated with DMSO or 8 μM of EI-52 for sixteen hours. Caspase-8 cleavage, and expression of p62 and tubulin were determined by western blot. Representative data of three independent experiments. **D** HCT116 cells were transfected with *p62* or Control siRNA for 24 h. Then, cells were treated with DMSO or 8 μM of EI-52 for 16 h, and ubiquitin aggregates were visualized by immunofluorescence staining. The bar represents 20 μm. Representative data of three independent experiments.

The ISR is a signaling cascade activated to support the maintenance of protein homeostasis in response to diverse cellular stressors [11–13]. In neurons, it was reported that ISR can lead to protein aggregation and eIF2α-dependent apoptosis, leading to neurodegenerative disorders [14]. We therefore investigated whether, in cancer cells, ISR induction and eIF2α activation by EI-52 also result in protein aggregate formation. Indeed, immunofluorescence staining showed that inhibition of the MyD88-ERK interaction by EI-52 induces the formation of cytoplasmic aggregates containing ubiquitinated proteins and the ubiquitin-binding protein p62, which progressively accumulate over time (Fig. 2A). Since cytoplasmic aggregates are insoluble [15], we performed fractionation experiments to separate cytosolic from insoluble proteins following treatment with EI-52. As expected, we observed an accumulation of p62 and ubiquitin in the insoluble fraction. Interestingly, cleaved caspase-8 was exclusively detected in this fraction in EI-52-treated, but not in untreated, cells, suggesting the presence of an apoptotic signaling platform within these aggregates (Fig. 2B and Supplementary Fig. 2A). Jin et al. reported that p62-mediated aggregation of polyubiquitinated caspase-8 acts as a crucial positive regulator of extrinsic apoptosis by amplifying caspase-8 activation [16]. However, unlike the mechanism triggered by EI-52, this process requires a surface signal mediated by FADD [16]. To further rule out the possibility that EI-52-induced caspase-8 activation involves a similar p62-dependent amplification loop, we silenced p62 expression using siRNA and then treated the cells with EI-52. Caspase-8 cleavage (Fig. 2C) and ubiquitin aggregate formation (Fig. 2D) persisted even in the absence of p62 (Supplementary Fig. 2B). Together, these results indicate that, when the ERK-MyD88 interaction is disrupted, caspase-8 activation occurs through an aggregation-driven mechanism that is independent of both FADD and p62. This finding uncovers a previously unrecognized, non-canonical pathway of caspase-8 activation.

### Inhibition of ERK-MyD88 interaction promotes myddosome assembly and chemokine production

Our previous data indicate that blocking the ERK-MyD88 interaction triggers NF-κB activation and chemokine release, driving the immunogenicity of cancer cell death [6]. In infection, TLR/IL-1R activation similarly induces NF-κB signaling through MyD88 oligomerization and recruitment of IRAK1 and IRAK4, leading to Myddosome assembly *via* Death Domain-mediated homotypic interactions. MyD88 overexpression has also been shown to induce MyD88 self-aggregation and downstream signaling independently of surface receptor activation [17]. We therefore hypothesized that disrupting the ERK-MyD88 interaction could liberate a pool of MyD88, promoting its aggregation, Myddosome assembly, and subsequent inflammatory signaling.

**Fig. 3:**
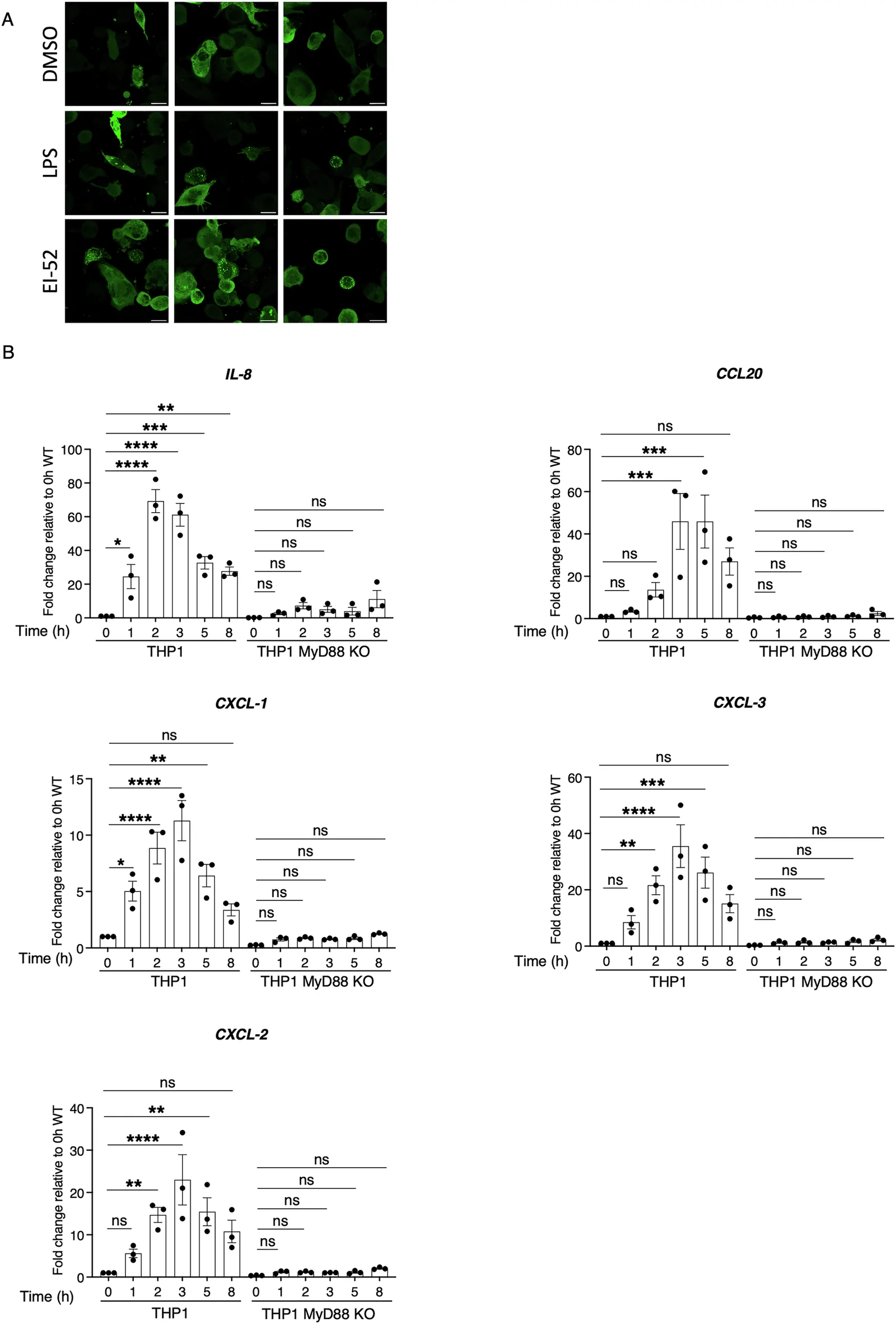
MyD88 oligomerizes upon EI-52 treatment, leading to chemokine production. **A** Confocal imaging of differentiated THP1-AGF treated for 1.5 h with LPS 2 μg/ml or 5 h with EI-52 10 μM. The bar represents 20 μm. Representative data of three independent experiments. **B** Chemokine mRNA from THP1 and THP1 MyD88 KO cells treated for the indicated time with EI-52 8 μM. Mean ± SEM from three independent experiments. Tukey’s one-way ANOVA IL-8 EI-52 0 vs 1 h *p = 0.0182, 0 vs 2 h ****p < 0.0001, 0 vs 3 h ****p < 0.0001, 0 vs 5 h ***p = 0.006, 0 vs 5 h **p = 0.0051; CCL20 EI-52 0 vs 3 h ***p = 0.0004, 0 vs 5 h ***p = 0.0005; CXCL-1 EI-52 0 vs 1 h *p = 0.0441, 0 vs 2 h ****p < 0.0001, 0 vs 3 h ****p < 0.0001, 0 vs 5 h **p = 0.0024; CXCL-3 EI-52, 0 vs 2 h **p < 0.0041, 0 vs 3 h ****p < 0.0001, 0 vs 5 h ***p = 0.0004; CXCL-2 EI-52, 0 vs 2 h **p < 0.0079, 0 vs 3 h ****p < 0.0001, 0 vs 5 h **p = 0.0044.

Indeed, among the proteins that accumulate in the insoluble fraction upon EI-52 treatment, we identified MyD88, IRAK1, and IRAK4 (Fig. 2B), which collectively constitute the core of the insoluble Myddosome signaling platform. To investigate Myddosome formation in response to EI-52, we used AGF-THP1 cells, which are CRISPR/Cas9-engineered to express endogenous MyD88 tagged at the C-terminus with Apex-GFP and Flag [18]. As expected, treatment with LPS for 1.5 h induced Myddosome formation, visualized as GFP clusters by confocal microscopy. Importantly, EI-52 treatment for 5 h also triggered assembly of Myddosomes, consistent with the idea that releasing MyD88 from its interaction with ERK enables its oligomerization into signaling complexes (Fig. 3A).

MyD88 activation via the Myddosome typically results in the production of chemokines, raising the possibility that the expression of these biological mediators during EI-52-induced immunogenic cell death also depends on MyD88 signaling. We had previously shown that the EI-52-induced chemokine signature includes *CXCL8*, *CXCL*1, *CXCL2*, *CXCL3*, and *CCL20* [6]. qPCR analysis revealed that EI-52 treatment markedly increased chemokine mRNA levels in WT THP-1 cells, whereas only marginal induction was observed in MyD88-deficient THP-1 cells (Fig. 3B and Supplementary Fig. 3A). These findings demonstrate that MyD88 is required for the induction of this chemokine signature in response to EI-52. By contrast, p62 inhibition by siRNA did not affect IL-8 secretion upon EI-52 treatment, suggesting that p62 is not involved in the regulation of this inflammatory response pathway triggered by EI-52 (Supplementary Fig. 3B).

**Fig. 4:**
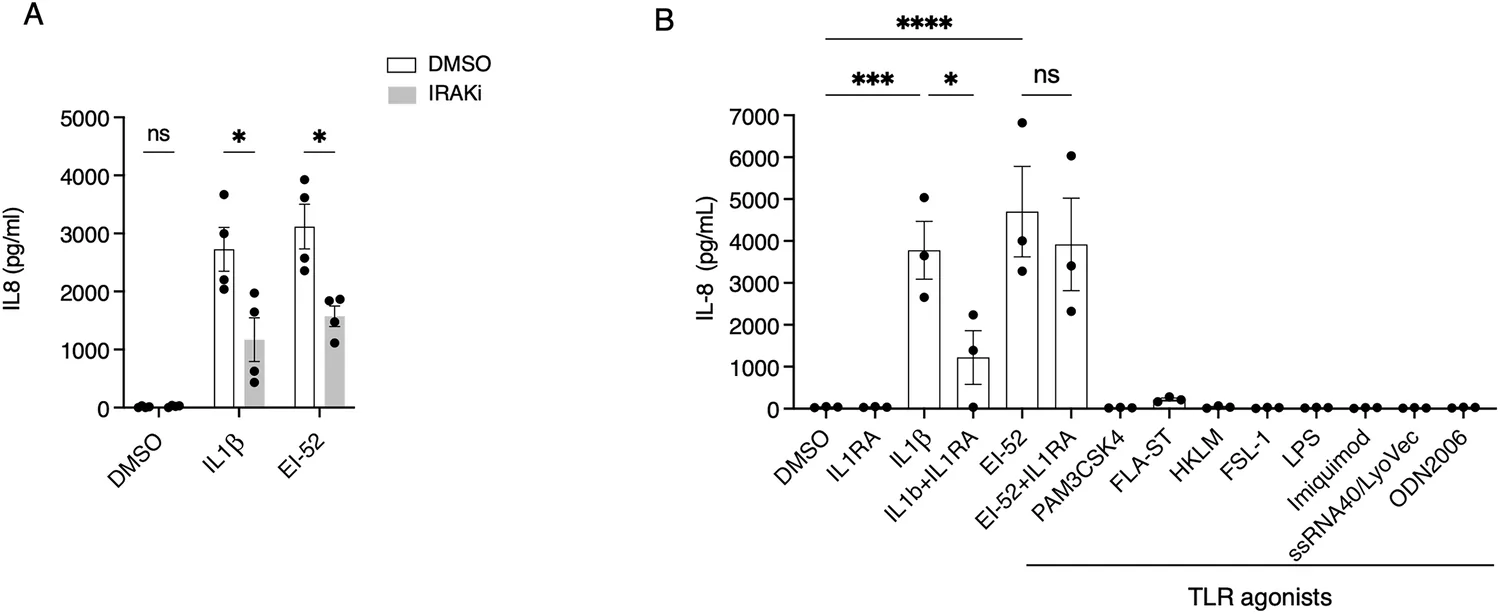
ERK-MyD88 interaction inhibition induces MyD88 signaling independently of the inflammatory receptors. **A** HCT116 cells were pre-incubated or not with IRAKi 10 μM for 1 h, then treated with IL1β 30 ng/ml or EI-52 8 μM for 16 h. Supernatants were collected and IL-8 quantified by ELISA. Mean ± SEM from four independent experiments. Šídák’s two-way ANOVA EI-52 (DMSO vs IRAKi) *p = 0.0341, IL-1β (DMSO vs IRAKi) *p = 0.0330, DMSO (DMSO vs IRAKi) ns *p* < 0.05. **B** HCT116 cells were pre-incubated or not with IL1-RA 1 μg/ml for 1 h prior to stimulation with IL1β 30 ng/ml or EI-52 10 μM. In parallel, cells were also treated with TLR agonists, including PAM3CSK4 10 ng/ml, FLA-ST 1 μg/ml, HKLM 10^7^–10^8^ cells/ml, FSL-1 100 ng/ml, LPS 1 μg/ml, Imiquimod 1 μg/ml, ssRNA40/Lyovec 1 μg/ml, ODN2006 5 μM for 16 h. Supernatants were collected and IL-8 quantified by ELISA. Mean ± SEM from 3 independent experiments Tukey’s one-way ANOVA DMSO vs EI-52 ****p < 0.0001 EI-52 vs EI-52 + IL1RA ns *p* < 0.05 DMSO vs IL1beta ***p = 0.0005 IL-1beta vs IL-1beta + IL1RA *p = 0.0413, DMSO vs IL1RA ns *p* < 0;05.

To formally demonstrate the role of MyD88 in EI-52-induced chemokine expression via Myddosome signaling, HCT116 cells were pretreated with an IRAK1/4 inhibitor for 1 h, then treated with either IL-1β or EI-52 for 16 h. IL-8 production was measured by ELISA. IL-1 β induces IL-8 secretion, which was partially blocked in the presence of the IRAK1/4 inhibitor. Similarly, EI-52 treatment triggered IL-8 secretion, which was suppressed to a similar extent by IRAK1/4 inhibition (Fig. 4A). These results further support the involvement of Myddosome-dependent signaling in chemokine induction following disruption of the ERK-MyD88 interaction.

### Chemokine production upon ERK-MyD88 disruption occurs independently of TLR/IL-1R signaling

Studies have shown that MyD88 oligomerization and myddosome formation depend on the amount of MyD88 available in the cell [19]. Consistent with this, our data suggest that EI-52 treatment disrupts the MyD88-ERK interaction, releasing a pool of free MyD88. The increase in free MyD88 may allow the protein to reach a threshold concentration needed for spontaneous Myddosome assembly. In particular, the accumulation of MyD88, IRAK1, and IRAK4 in the insoluble fraction following EI-52 treatment suggests that chemokine production is likely independent of TLR and IL-1R activation. To rule out a contribution from surface receptors, we treated HCT116 cells, which express a limited number of TLRs, with a panel of agonists that activate the TLRs that signal via MyD88. As expected, none of the TLR agonists induced IL-8 production in these cells, thus ruling out MyD88 activation by TLR ligands upon EI-52 treatment (Fig. 4B). In contrast, IL-1β did induce IL-8 production to a similar extent as EI-52. However, whereas pre-treatment with the IL-1 receptor antagonist (IL-1RA) markedly reduced IL-1β-induced IL-8 production, it had no significant effect on EI-52-induced IL-8 secretion.

Altogether, these results demonstrate that EI-52-induced chemokine production depends on MyD88 signaling and support the hypothesis that MyD88, once released from its interaction with ERK, drives spontaneous Myddosome assembly independent of canonical receptor-mediated pathways. This interpretation is consistent with previous observations showing that MyD88 overexpression or the oncogenic L265P mutation can activate NF-κB signaling in the absence of IL-1 or TLR ligand stimulation [20,21].

Collectively, our data suggest that disrupting the interaction between ERK and MyD88 can, independently of surface receptors, trigger the aggregation of unbound MyD88, leading to Myddosome formation and the activation of inflammatory signaling. Although the exact molecular mechanisms underlying MyD88 release from ERK and the role of aggregates in caspase-8 activation remain to be fully elucidated, our findings indicate that perturbing ERK protein-protein complexes, rather than inhibiting its kinase activity, can activate non-canonical signaling pathways. Exploiting such receptor-independent pathways that promote immunogenic cell death through the assembly of alternative signaling hubs could hold substantial promise for innovative cancer therapies.

## Discussion

Our results reveal that disrupting the ERK-MyD88 interaction engages a dual signaling program that combines apoptosis with immunostimulatory activity. On one hand, EI-52 activates caspase-8 through a non-canonical, aggregation-driven mechanism that bypasses both FADD and p62. On the other hand, ERK-MyD88 disruption liberates MyD88 to form Myddosomes, thereby promoting NF-κB activation and chemokine secretion. Together, these findings highlight a receptor-independent mechanism of cancer cell death with potential immunogenic consequences.

The identification of caspase-8 activation independent of FADD is particularly noteworthy. Although previous studies have reported non-canonical caspase-8 engagement in response to chemotherapeutic agents or calcium flux [22, 23], this represents, to our knowledge, the first demonstration of such a mechanism elicited by a targeted therapy. The localization of cleaved caspase-8 within the insoluble aggregate fraction is intriguing, as one might expect active caspase-8 to be released into the cytosol in order to propagate downstream apoptotic signaling. However, several studies have reported the presence of active caspase-8 within protein aggregates or higher-order complexes, suggesting that aggregates may serve not only as sequestration sites but also [24, 25]. In contrast to previously described aggregate-dependent mechanisms of caspase-8 activation, EI-52 triggers caspase-8 activation in the absence of p62, highlighting the distinct nature of this pathway. Notably, FADD-independent caspase-8 activation in p62-silenced cells is particularly striking, as ubiquitinated protein aggregates are conventionally organized by p62, which bridges ubiquitinated substrates to autophagic membranes and facilitates caspase-8 [16, 25, 26].

In parallel, ERK-MyD88 disruption initiated MyD88 oligomerization and Myddosome assembly independently of TLR or IL-1R ligands. Similar to the L265P oncogenic MyD88 mutation, this is the first evidence that a targeted therapy can induce MyD88 oligomerization independently of receptor engagement. The resulting chemokine release suggests that disrupting ERK-MyD88 complexes may enhance the immunogenicity of tumor cell death and promote the recruitment of immune effectors.

Importantly, these effects are not attributable to inhibition of ERK kinase activity, but rather to disruption of ERK’s scaffolding function. This distinction emphasizes that protein-protein interaction targeting can elicit signaling outcomes inaccessible to classical kinase inhibitors. By rewiring both apoptotic and inflammatory pathways, ERK-MyD88 blockade represents a promising strategy to couple tumor cell killing with immune activation. Future work should further define the molecular composition of the aggregates driving caspase-8 activation and explore how this pathway can be exploited for therapeutic gain, either alone or in combination with immunotherapies.

## Material and methods

### Cell lines and reagents

Human HCT116 (RRID: CVCL_0291) colorectal cancer cells were purchased from ATCC. Jurkat and Jurkat-FADD KO were kindly provided by O. Micheau (INSERM, Dijon). THP1, THP1-MyD88-AGF and THP1 MyD88 KO were kindly provided by J.C. Kagan (Boston Children’s Hospital) and HCT116_SCR and HCT116_CASP8_KO were kindly provided by D.B. Longley (Queen’s University Belfast). The absence of mycoplasma contamination in the cell lines is regularly tested using the MycoAlert Mycoplasma Detection Kit (Lonza). eIF2a inhibitor (ISRIB) and IRAK1/4 inhibitor I were purchased from Selleckchem, necrosulfamide from Tocris, SQSTM1 (p62) (L-010230) ON-TARGETplus SMARTpool human siRNAs, RIPK1 no. 7 (J-004445-07), RIPK1 no. 8 (J-004445-08) human siRNAs from Horizon Discovery, human Caspase 8 (SI02661946) and FADD (SI03649016) from Qiagen, and non-targeting siRNA from Eurofin Genomics. TLRs agonists and IL1β were from INVIVOGEN. IL1RA was purchased from Biotechne.

Antibodies used are listed in Table 1.

**Table 1:** Antibodies.

Antibody	Company	Reference	RRID	Species	Dilution	Application
Ubiquitin	ENZO	ENZABS840	RRID:AB_3714780	Mouse	1/500	IF
P62	ABCAM	Ab109012	RRID:AB_2810880	Rabbit	1/500	IF
anti-mouse Alexa Fluor 633	Invitrogen	A21070	RRID:AB_2535731	Goat	1/500	IF
anti-mouse Alexa Fluor 488	Invitrogen	A11001	RRID: AB_2534069	Goat	1/500	IF
anti-rabbit Alexa Fluor 488	Invitrogen	A11034	RRID: AB_2576217	Goat	1/500	IF
Caspase 8	CST	9746	RRID:AB_2275120	Rabbit	1/1000	WB
Caspase 9	R&D	MAB8301	RRID:AB_2073457	Rabbit	1/1000	WB
P62	Genetex	GTX629890	RRID:AB_2885144	Mouse	1/1000	WB
Ubiquitin	ENZO	BML-PW8810	RRID:AB_10541840	Mouse	1/1000	WB
MyD88	CST	4283	RRID:AB_10547882	Rabbit	1/1000	WB
IRAK1	CST	4504	RRID:AB_1904032	Rabbit	1/1000	WB
IRAK4	CST	4363	RRID:AB_2126429	Mouse	1/20 000	WB
FADD	CST	2782	RRID:AB_2100484	Mouse	1/10 000	WB
p-eIF2a	CST	3398	RRID:AB_2096481	Rabbit	1/1000	WB
ATF4	CST	11815S	RRID:AB_2616025	Rabbit	1/1000	WB
Actin	Sigma	A5441	RRID:AB_476744	Mouse	1/1000	WB
Tubulin	Sigma	T6199	RRID:AB_477583	Rabbit	1/3 000	WB
RIPK1	CST	4926	RRID:AB_2224503	Rabbit	1/1000	WB

### Apoptosis detection

For apoptosis detection, 2 × 10^5^ HCT116 cells were treated for 8 h with DMSO or 8 or 12 μM EI-52, then stained with AnnexinV-fluorescein isothiocyanate (FITC) and propidium iodide (Apoptosis Detection Kit, Abcys). Fluorescence was acquired on a BD FACScalibur and analyzed using FlowJo software.

### Cell death

1.5 × 10^5^ HCT116 cells were seeded onto 96-well plates. The cells were then incubated with EI-52 in the presence or absence of inhibitors, in medium containing propidium iodide at 3.5 μg/mL. The percentage of dead cells was determined using the IncuCyte^™^ Kinetic Live Cell Imaging System (Essen BioScience) at 2 h intervals and up to 24 h.

### Protein extraction and Western blot

Cell fractionation: HCT116 cells were treated for 4 and 16 h with DMSO or 8 μM EI-52. Briefly, cells were washed twice with PBS 1X and resuspended with hypotonic buffer (10 mM HEPES pH8, 10 mM KCl, 0.1 mM EDTA, 0.5% NP-40, 1 mM DTT added extemporaneously) with protease and phosphatase inhibitors on ice. Cells were examined under a microscope to confirm that the cytoplasm had been lysed while the nuclei remained intact, and then centrifuged at 12,000 × *g* for 5 min. The supernatant was collected as the cytoplasm fraction. The pellet was washed with hypotonic buffer and lysed with hypertonic buffer (20 mM HEPES pH8, 400 mM NaCl, 0.1 mM EDTA, 0.5% NP-40, 1 mM DTT added extemporaneously) with protease and phosphatase inhibitors on ice to prepare the nuclear fraction. Lysates were sonicated briefly and centrifuged at 10,000 g for 15 min at 4 °C. The remaining pellet is considered as the insoluble fraction, was washed with hypertonic buffer, and was lysed in 2% SDS containing protease and phosphatase inhibitors, and sonicated briefly. Protein concentrations were determined using the BCA assay (Pierce BCA Protein Assay Kit, Thermo Fisher Scientific; Cat. No. 23225).

Whole cell extracts: Cells were lysed in Laemmli buffer (125 mM Tris-HCL (pH 6.8), SDS 2%) and supplemented with a mixture of protease and phosphatase inhibitors. Lysates were sonicated and then centrifuged for 15 min at 15,000 × *g* at 4 °C.

Proteins were resolved on SDS- PAGE, transferred onto PVDF membranes by electroblotting, and nonspecific binding sites were blocked using Tris-buffered saline containing 5% (w/v) nonfat dry milk and 0.1% Tween. After incubation with primary antibodies overnight at 4 °C, membranes were rinsed, incubated with horseradish peroxidase-conjugated secondary antibody and revealed by chemiluminescence using Bio-Rad Clarity Western ECL and Bio-Rad Chemidoc.

### Immunofluorescence

HCT116 cells were treated as described in the legend. Then, cells were fixed with ice-cold methanol for 5 min then permeabilized with 0.1% Triton X-100 for 5 min and then blocked with 1% BSA/10% normal goat serum/0.3 M glycine in PBS-Tween 0.1%. Ubiquitin and p62 antibodies were incubated overnight at 4 °C, followed by a further incubation at room temperature for 1 h with secondary antibodies. Nuclear DNA was labeled in blue with DAPI, and fluorescence was observed by microscopy (Nikon).

### Chemokine mRNA quantification

THP1 or THP1 MyD88 KO cells were seeded onto 12-well plates. The next day, cells were treated with vehicle (DMSO) or 10 μM EI-52 or 1 μg/ml LPS for the indicated time. For qPCR, total cellular RNA was extracted using the NucleoSpin RNA Kit (Macherey-Nagel). Then 0.5 µg of RNA was reverse transcribed using the iScript Reverse transcriptase Supermix (Biorad) and quantified by real-time PCR using specific primers for *CXCL1, CXCL2, CXCL3* and *CXCL8* (Table 2) and the TB Green premix ExTaq TM II (Takara Bio). qPCR was performed using the CFX connect real-time PCR system (Biorad). Expression of target genes was normalized against endogenous RSPII mRNA levels, used as an internal control, and assessed using the comparative 2^–ΔΔCt^ method.

**Table 2:** Primer sequences.

Primer Name	Sequence
*CXCL1*-Fwd	TCCTGCATCCCCCATAGTTA
*CXCL1*-Rev	CTTCAGGAACAGCCACCAGT
*CXCL2*-Fwd	CCCATGGTTAAGAAAATCATCG
*CXCL2*-Rev	CTTCAGGAACAGCCACCAAT
*CXCL3*-Fwd	TGAATGTAAGGTCCCCCGGA
*CXCL3*-Rev	ACCCTGCAGGAAGTGTCAAT
*CXCL8*-Fwd	AGACAGCAGAGCACACAAGC
*CXCL8*-Rev	ATGGTTCCTTCCGGTGGT

### Myddosome imaging

Samples were imaged using the LSM 980 (Zeiss) on the Cell Imaging Platform (PIC, CRCL, Lyon) at 37 °C with moist 5% of CO2. Images were acquired with a Plan-Apochromat 63X/1.44 objective lens using the 488 nm excitation laser. GFP images were acquired simultaneously, respectively, with a GaAsp detector from 490 to 597 nm and a Multialkali detector. 5 images were taken to make a 16 µm stack at different timepoints with multiple fields per well.

### IL8 quantification

10^5^ HCT116 cells were seeded onto 12-well plates. The next day, cells were treated as indicated in the figure legend. ELISA was used to quantify secreted CXCL8 (Biolegend).

### Statistical and reproducibility

All the statistical analyses were performed on GraphPad Prism v.10 by using unpaired t-test, one- or two-way ANOVA, Wilcoxon, or Mann-Whitney. *P*-values < 0.05 were considered statistically significant and are represented by (*) symbols. Exact p-values are reported when provided by the software, whereas non-significant results are denoted ns.

No statistical method was used to predetermine sample size. Experiments were not randomized. The investigators were not blinded to allocation during experiments and outcome assessment. No data were excluded from the analysis.

## Supplementary information

Supplementary information

Uncropped WB

## Data Availability

All data needed to evaluate the conclusions in the article are present in the manuscript and or the Supplementary Materials.
