# Supplementary material for: Non-canonical signaling hubs upon inhibition of MyD88-ERK interaction in cancer
Source: Cell Death Dis. 2026 Jul 30;17(1):833. doi: 10.1038/s41419-026-09131-z (PMC13614936; doi:10.1038/s41419-026-09131-z)
Supplement: Supplementary file 1 [file 41419_2026_9131_MOESM1_ESM.pdf]

SUPPLEMENTARY INFORMATION

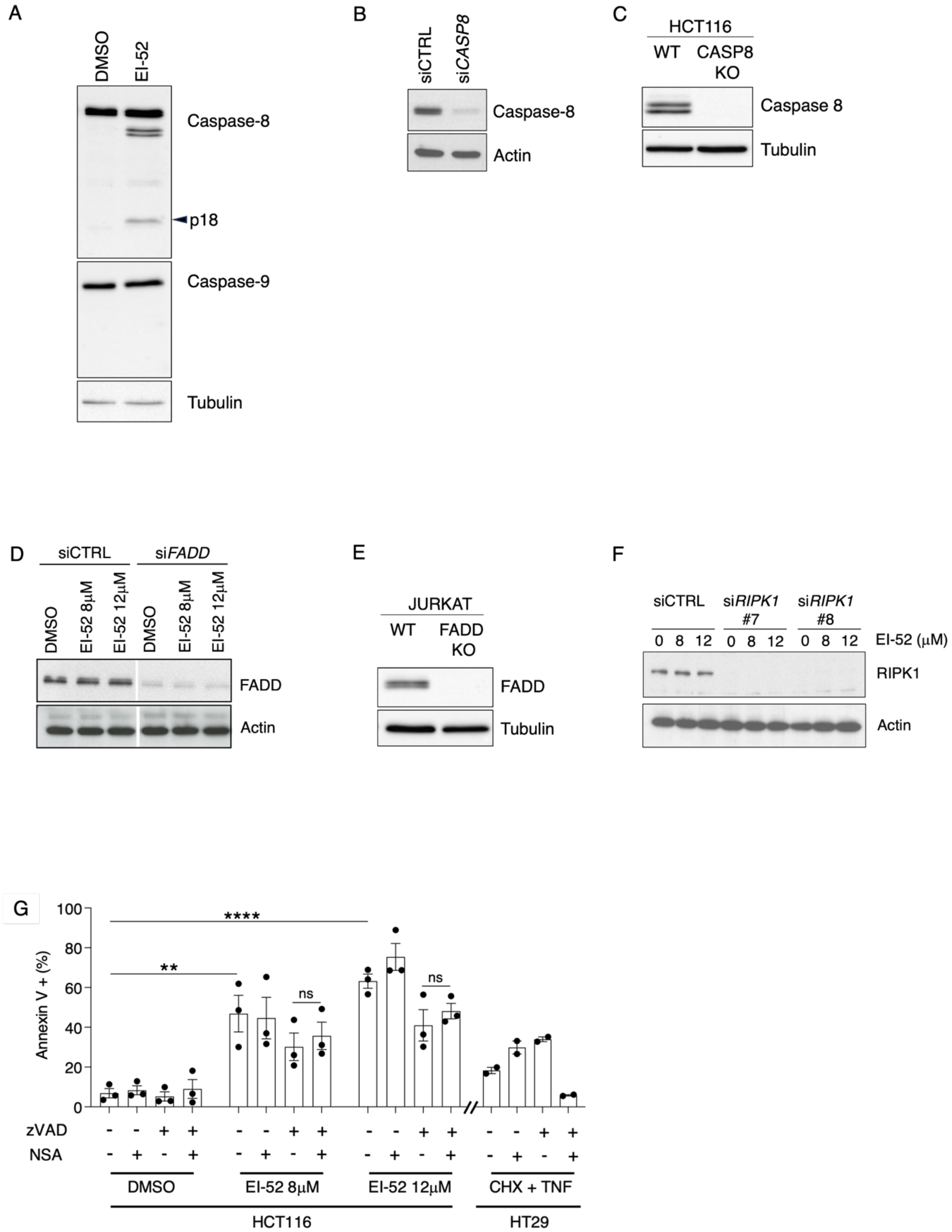

SUPPLEMENTARY FIGURE 1

(A) HCT116 cells were treated for 16 hours with DMSO or EI-52 8 μM. Caspase 8 and 9 cleavage was evaluated by western blot. Representative data of three independent experiments. **(B)** HCT116 cells were transfected with *Caspase 8* or Control siRNA for twenty-four hours. Expression of Caspase 8 and Actin was determined by western blot. Representative of three independent experiments. **(C)** Proteins were extracted from HCT116 or Caspase 8 KO cells and expression of Caspase 8 and Tubulin was determined by western blot. **(D)** HCT116 cells were transfected with *FADD* or Control siRNA for twenty-four hours. Expression of FADD and Actin was determined by western blot. Representative data of three independent experiment. **(E)** Proteins were extracted from JURKAT or JURKAT FADD KO cells and expression of FADD and Tubulin was determined by western blot. **(F)** HCT116 cells were transfected with *RIPK1#7 or #8* or Control siRNA for twenty-four hours. Expression of RIPK1 and Actin was determined by western blot. Representative of three independent experiments. **(G)** HCT116 cells were treated for twenty-four hours with DMSO or EI-52 at 8 or 12 μM in presence or absence of necrosulfamide (NSA) 1 μM, ZVAD 25 μM or a combination of both. HT29 cells were treated with cycloheximide (CHX) 5 μg/ml and TNFα 50ng/ml in presence or absence of necrosulfamide 1 μM, ZVAD 25 μM or a combination of both. Tukey's one-way ANOVA, DMSO vs EI-52 8 μM ** $p=0.005$, DMSO vs EI-52 12 μM **** $p<0.0001$, EI-52 8 μM + ZVAD vs EI-52 8 μM + ZVAD + NSA non-significant ns, EI-52 12 μM + ZVAD vs EI-52 12 μM + ZVAD + NSA non-significant ns (95% confidence interval). Representative data of three independent experiments

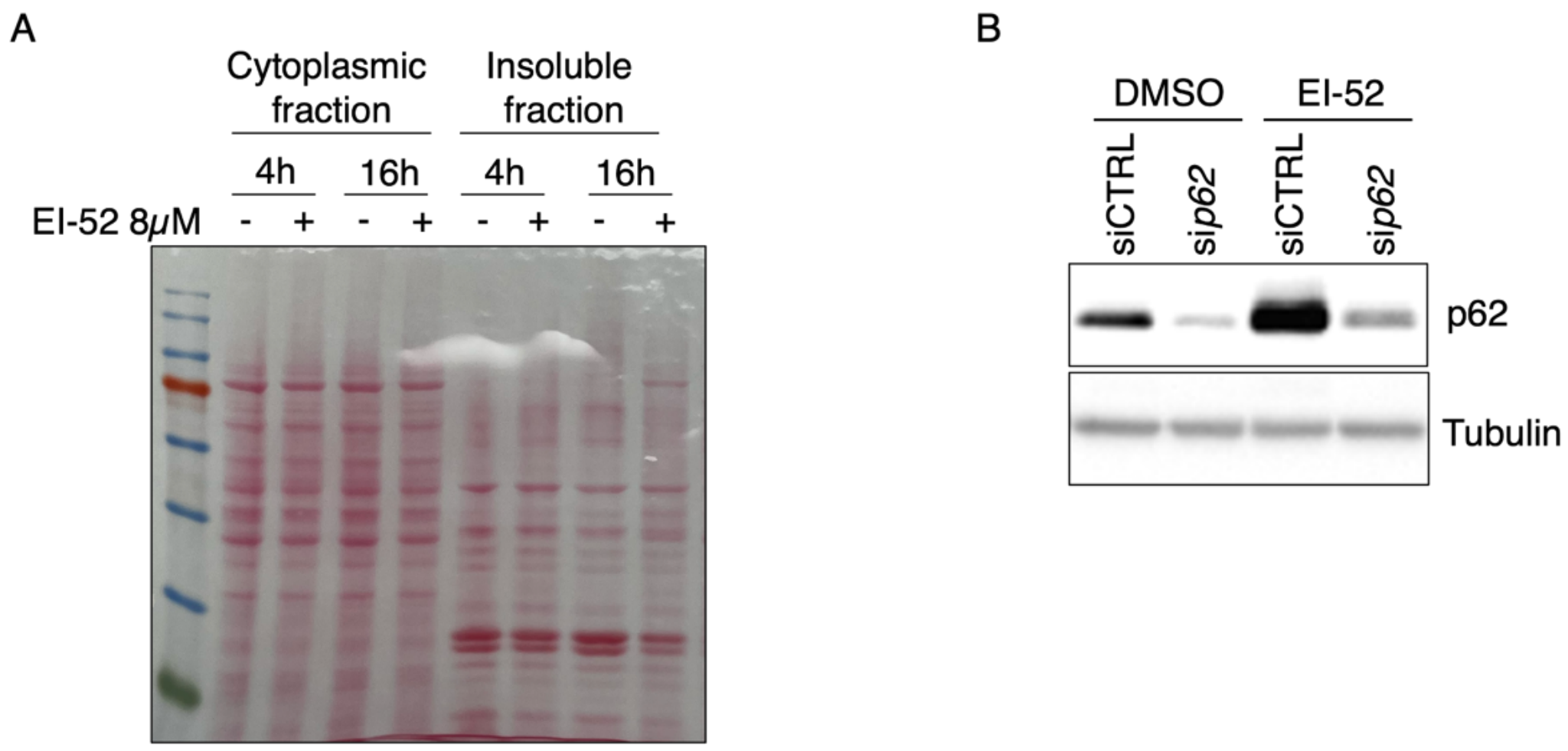

SUPPLEMENTARY FIGURE 2

(A) Ponceau Red staining was used as a loading control for the fractionation experiment. Representative data of three independent experiments. **(B)** HCT116 cells were transfected with *p62* or Control siRNA for twenty-four hours. Expression of p62 and tubulin was determined by western blot. Representative data of three independent experiments.

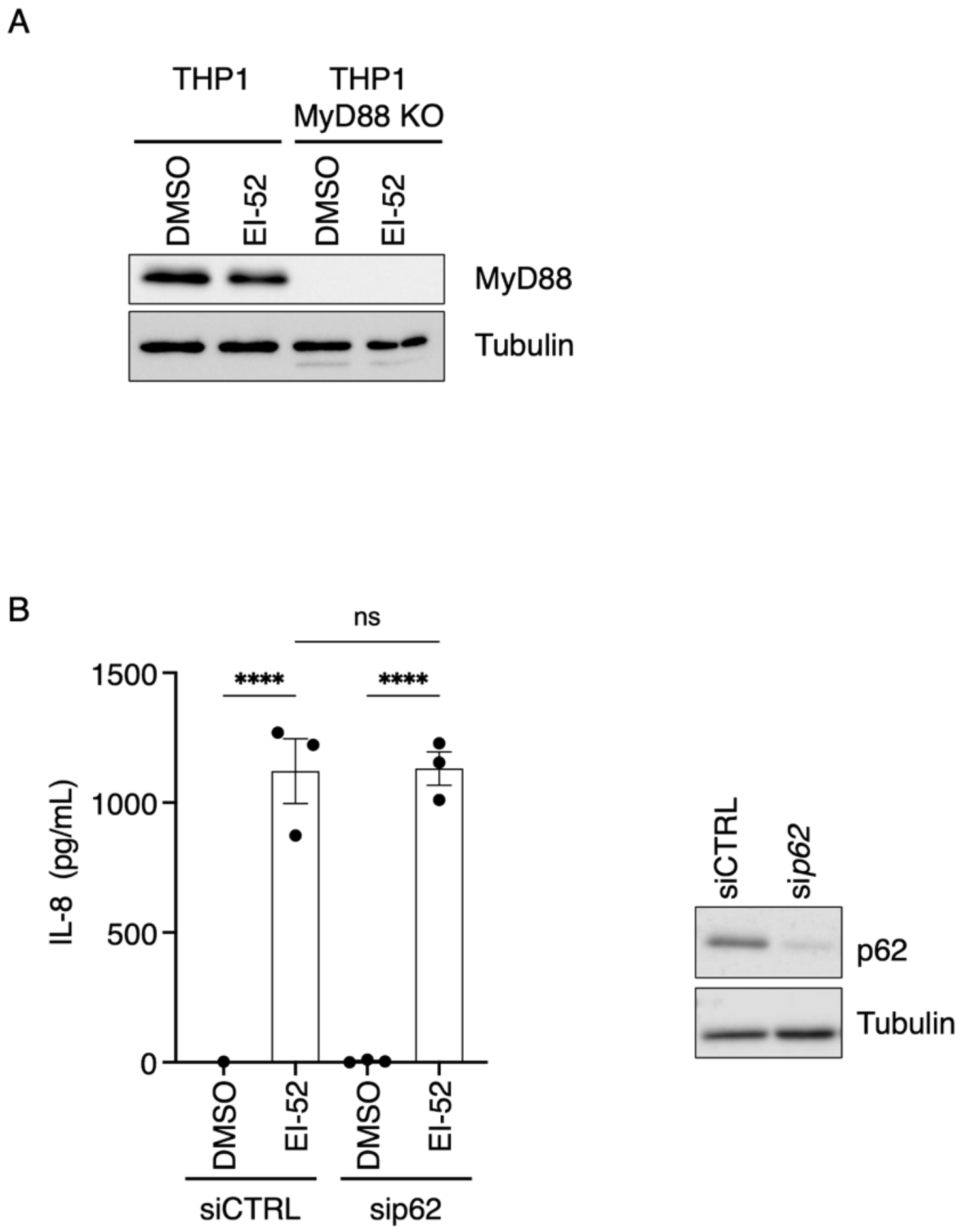

SUPPLEMENTARY FIGURE 3

(A) Proteins were extracted from THP1 or THP1 MyD88 KO cells and expression of MyD88 and Tubulin was determined by western blot. **(B)** HCT116 cells were transfected with *p62* or Control siRNA for twenty-four hours. Then cells were treated with DMSO or 8 µM of EI-52 for sixteen hours. Supernatants were collected and IL-8 quantified by ELISA (Left panel). Mean ± SEM from 3 independent experiments. Tukey's one-way ANOVA, siCTRL DMSO vs siCTRL EI-52 **** p<0.0001, si*p62* DMSO vs si*p62* EI-52) **** p<0.0001, siCTRL EI-52 vs si*p62* EI-52 non-significant ns. Expression of P62 and tubulin was determined by western blot (Right panel). Representative data of three independent experiments.
