# Supplementary material for: Non-canonical signaling hubs upon inhibition of MyD88-ERK interaction in cancer
Source: Cell Death Dis. 2026 Jul 30;17(1):833. doi: 10.1038/s41419-026-09131-z (PMC13614936; doi:10.1038/s41419-026-09131-z)

Figure 1a

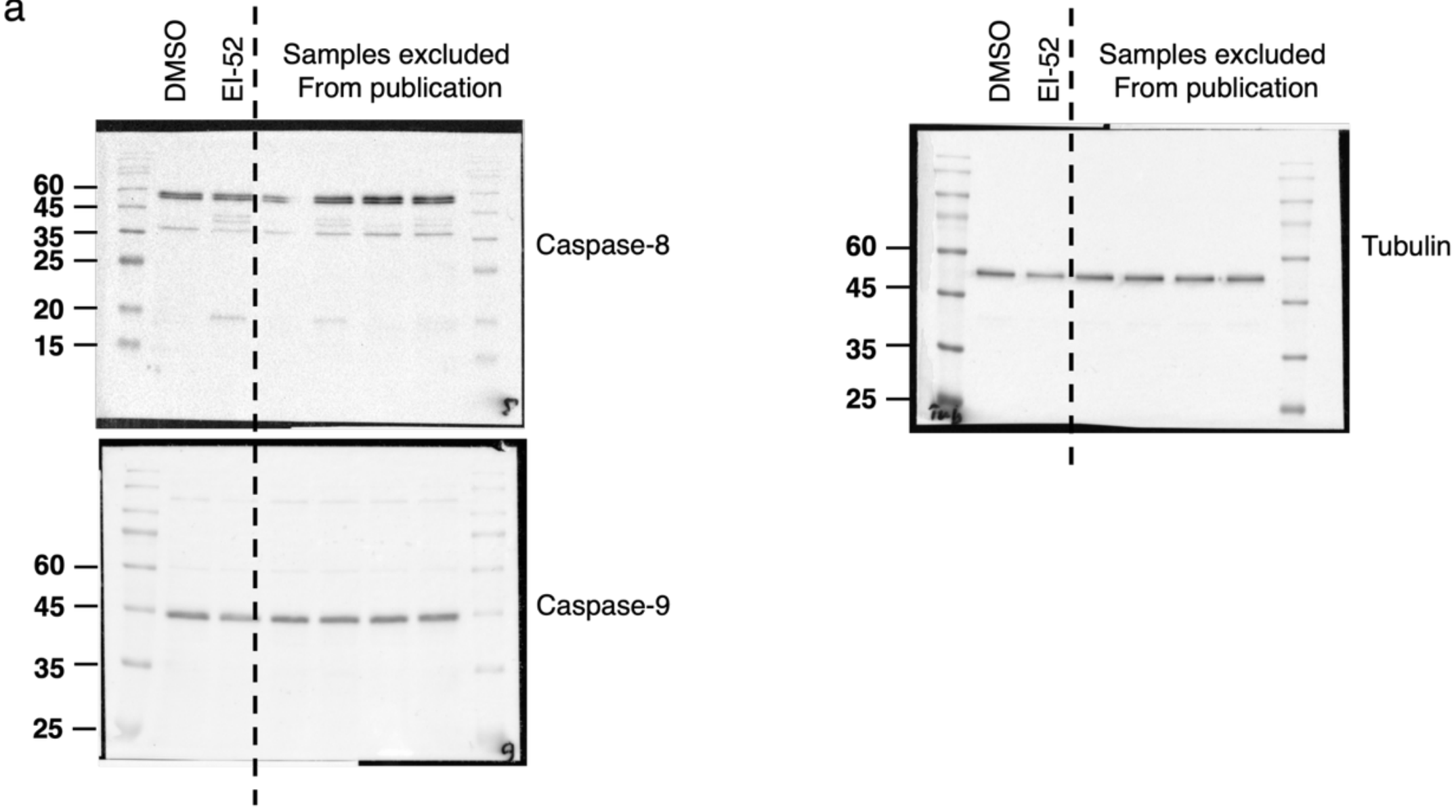

Figure 1g

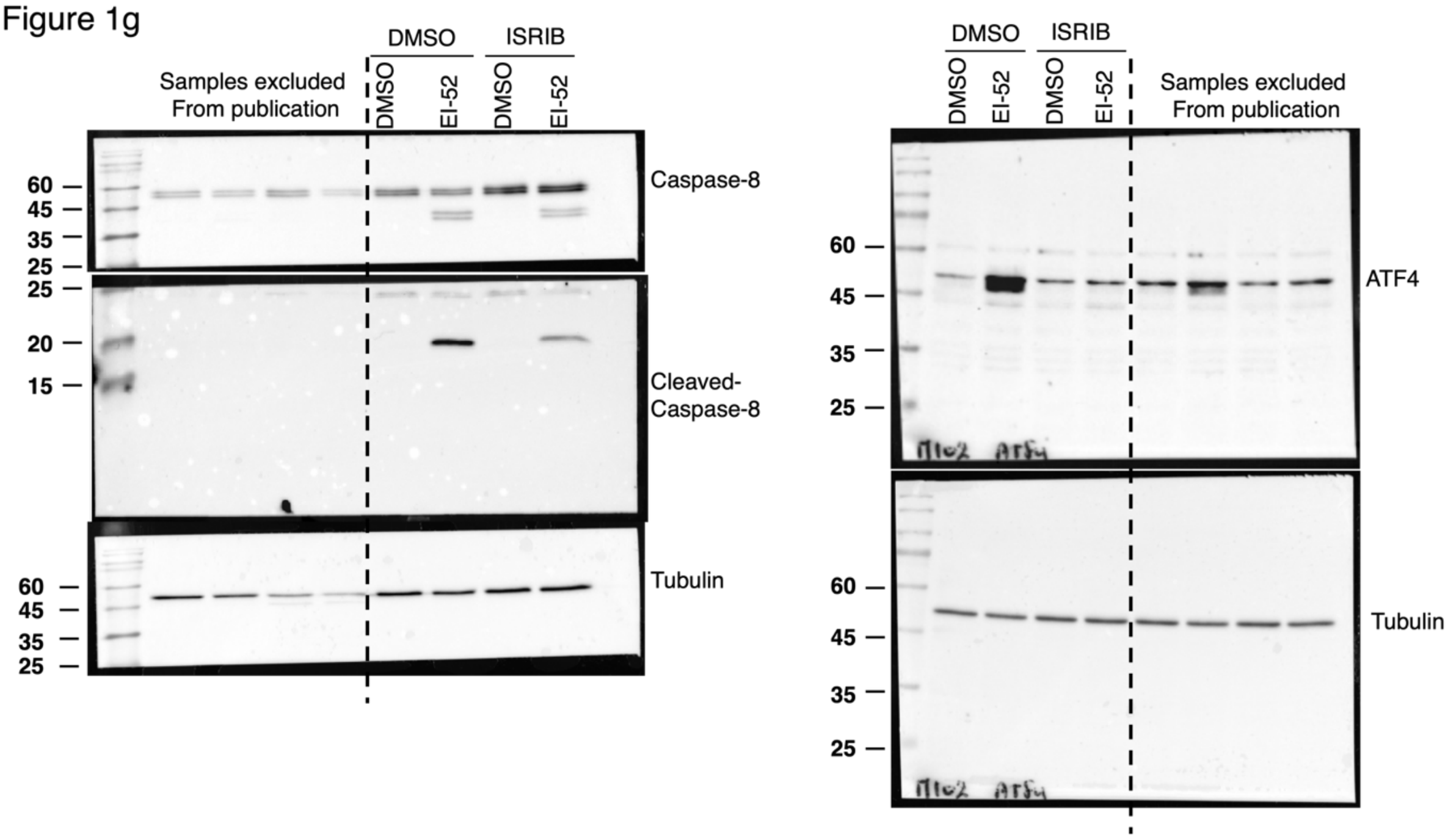

Figure 2b

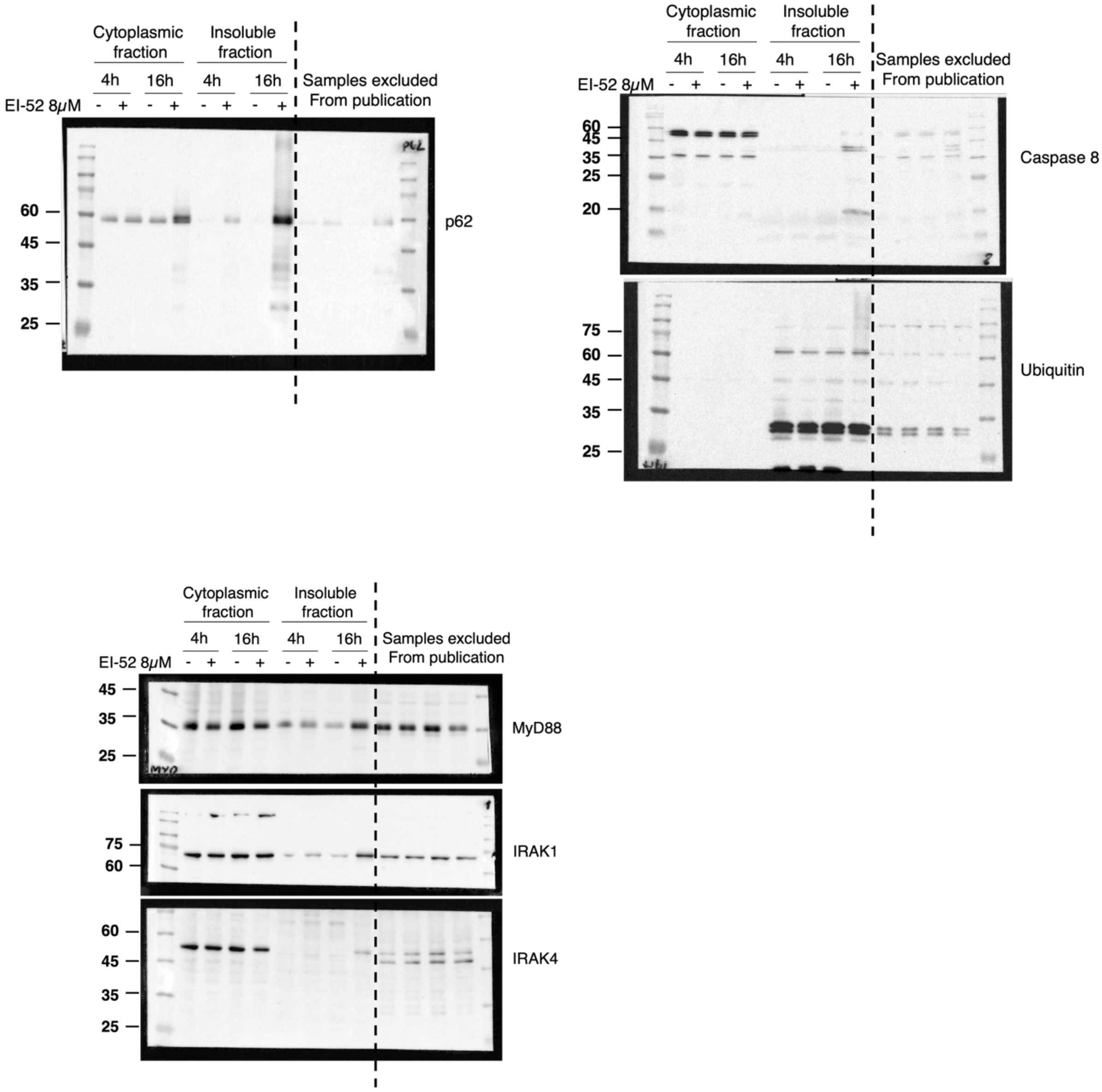

Cytoplasmic fraction
Insoluble fraction
4h
16h
EI-52 8μM
Samples excluded From publication
60
45
35
25
p62
20
Caspase 8
75
Ubiquitin
MyD88
IRAK1
IRAK4

Figure 2c

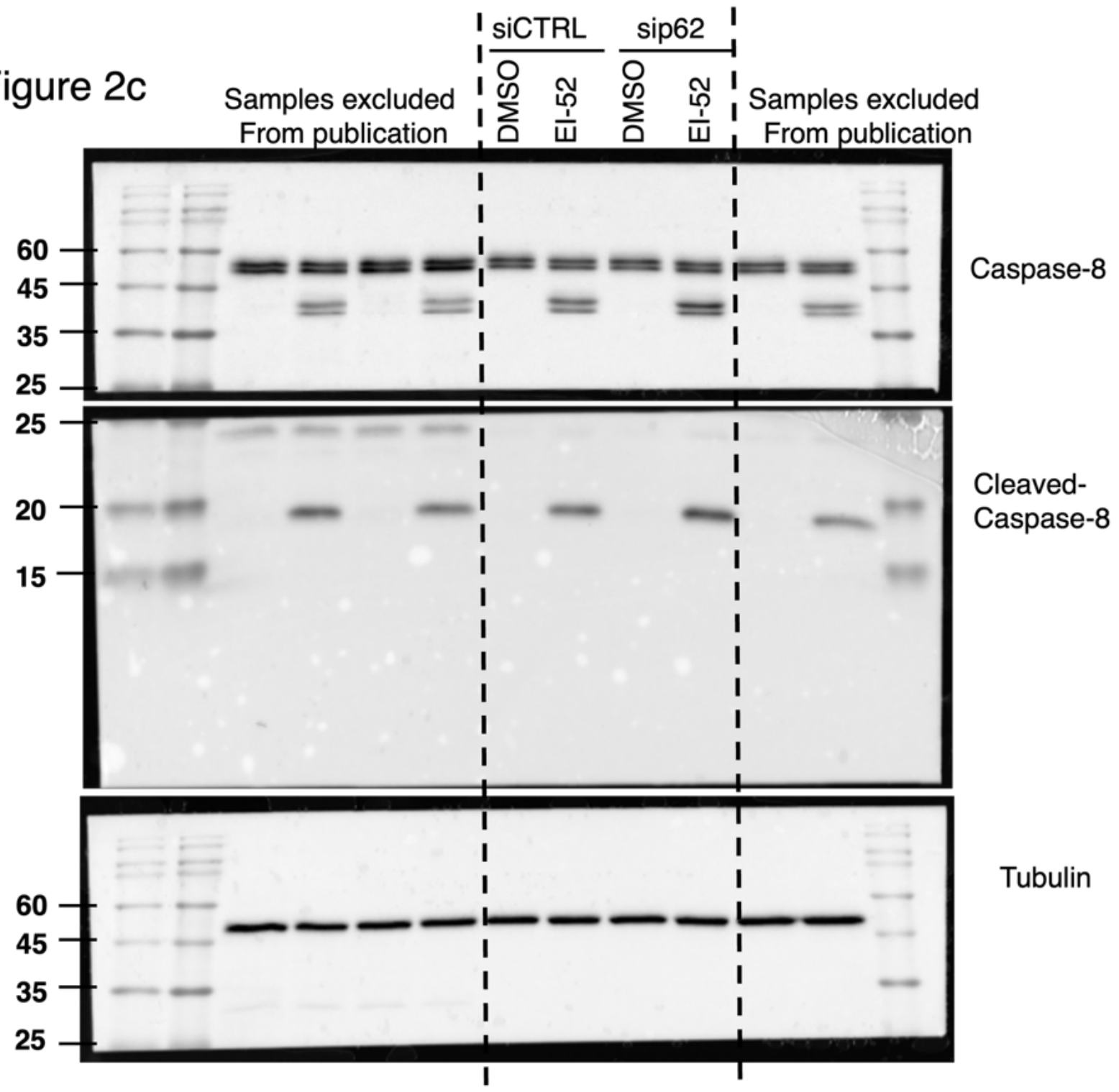
siCTRL
sip62
DMSO
EI-52
DMSO
EI-52
Samples excluded From publication
Samples excluded From publication
60
45
35
25
Caspase-8
25
20
15
Cleaved-Caspase-8
60
45
35
25
Tubulin

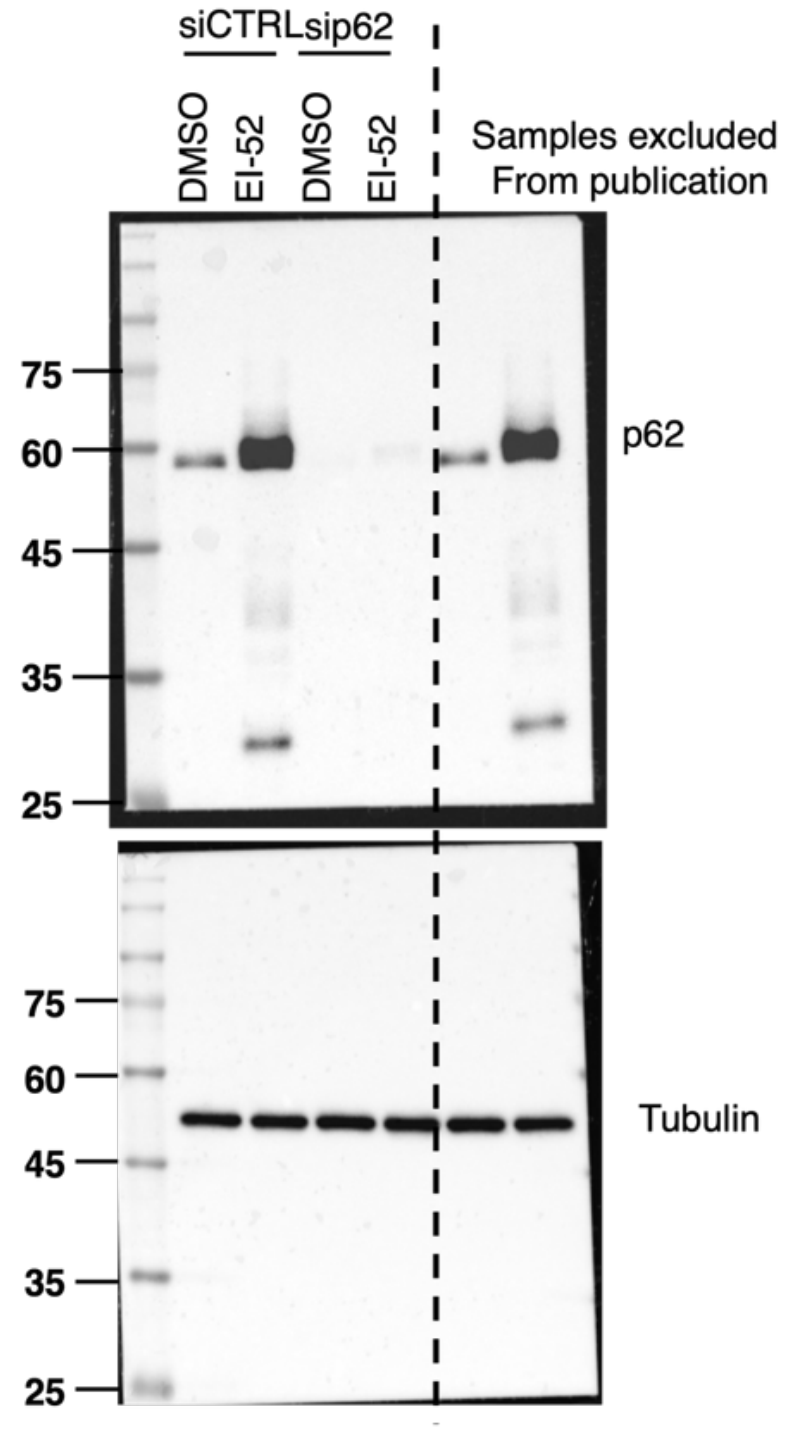
siCTRL
sip62
DMSO
EI-52
DMSO
EI-52
Samples excluded From publication
75
60
45
35
25
p62
75
60
45
35
25
Tubulin

Supplementary figure 1a

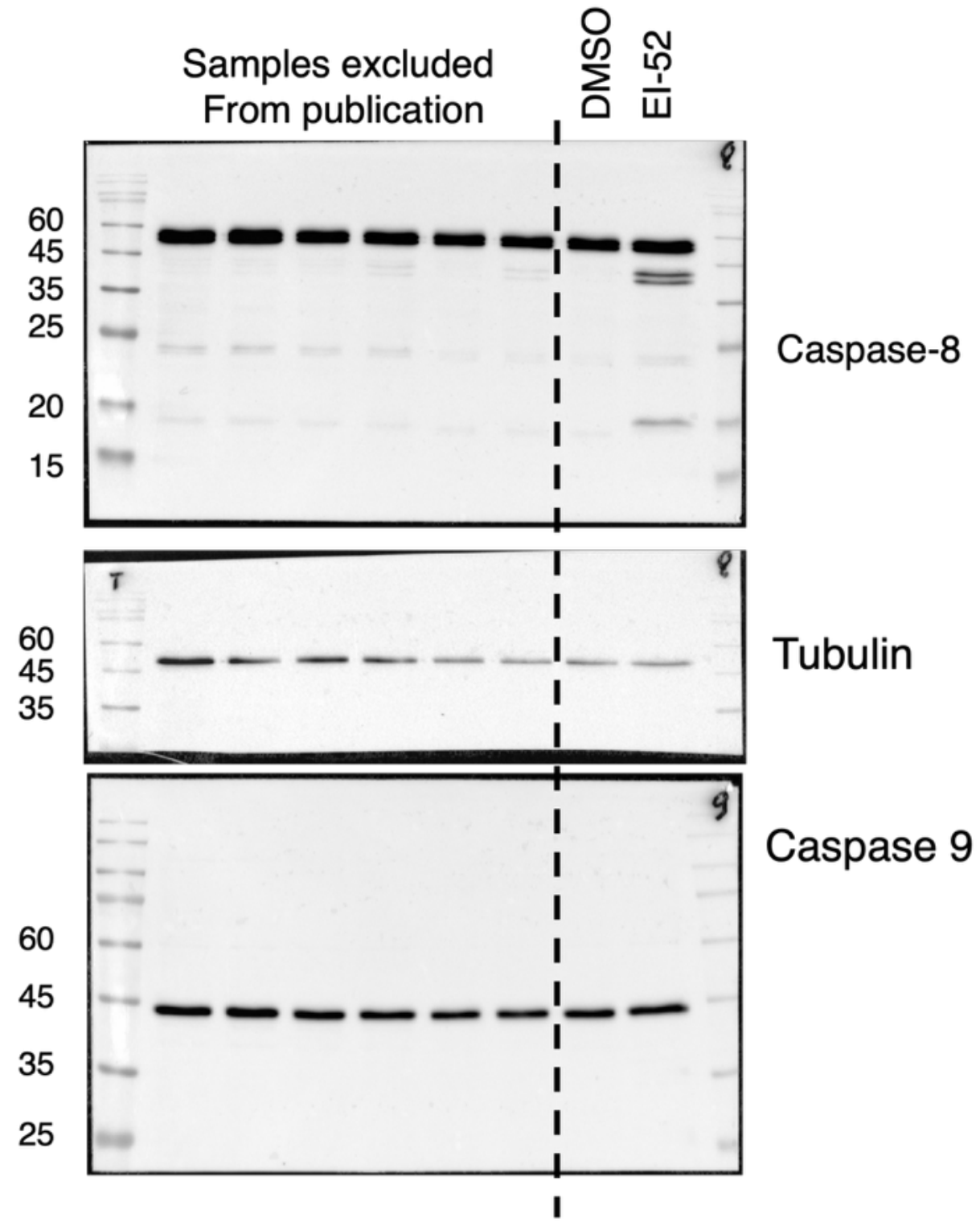

Supplementary Figure 1b

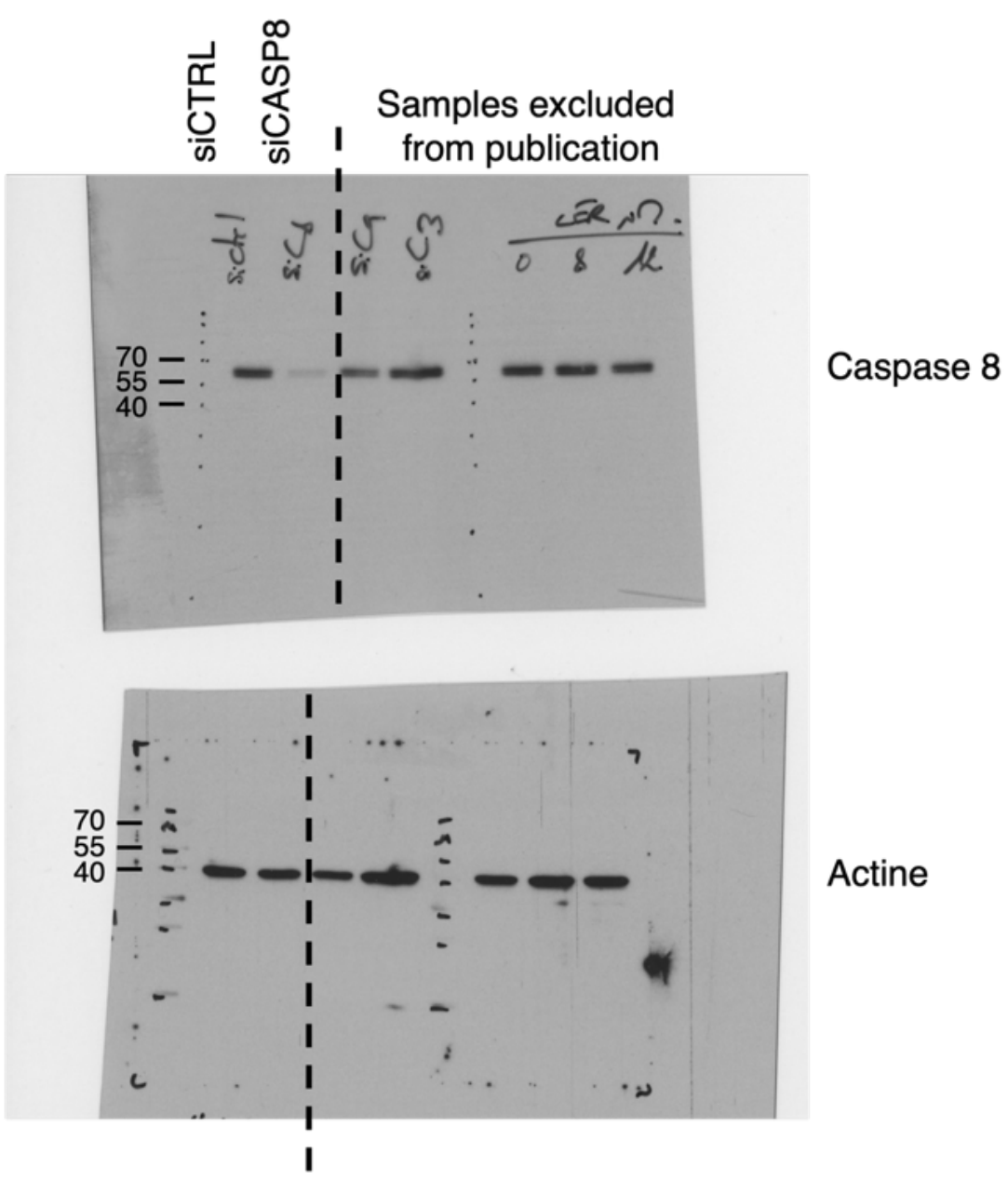

Supplementary Figure 1c

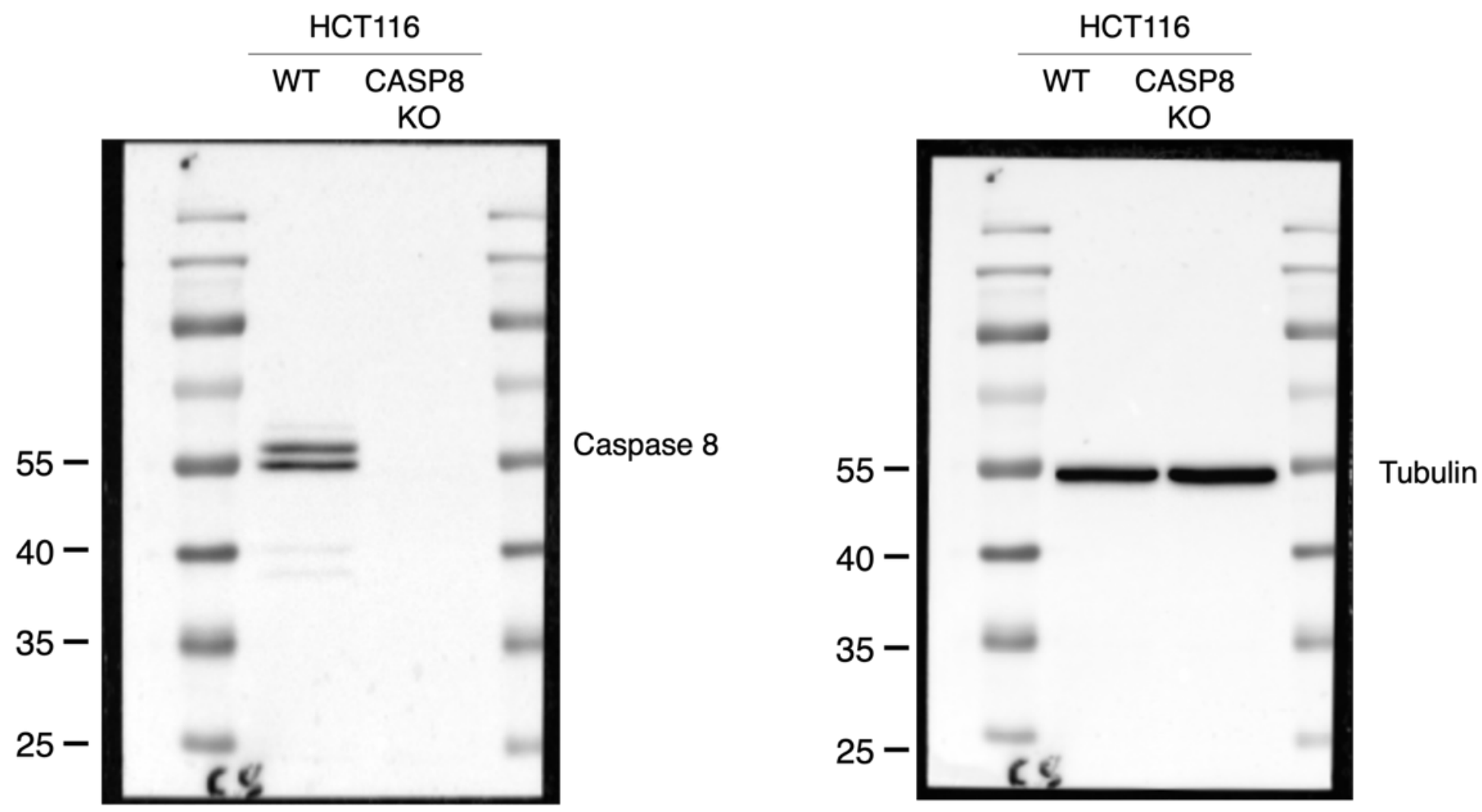

Supplementary Figure 1d

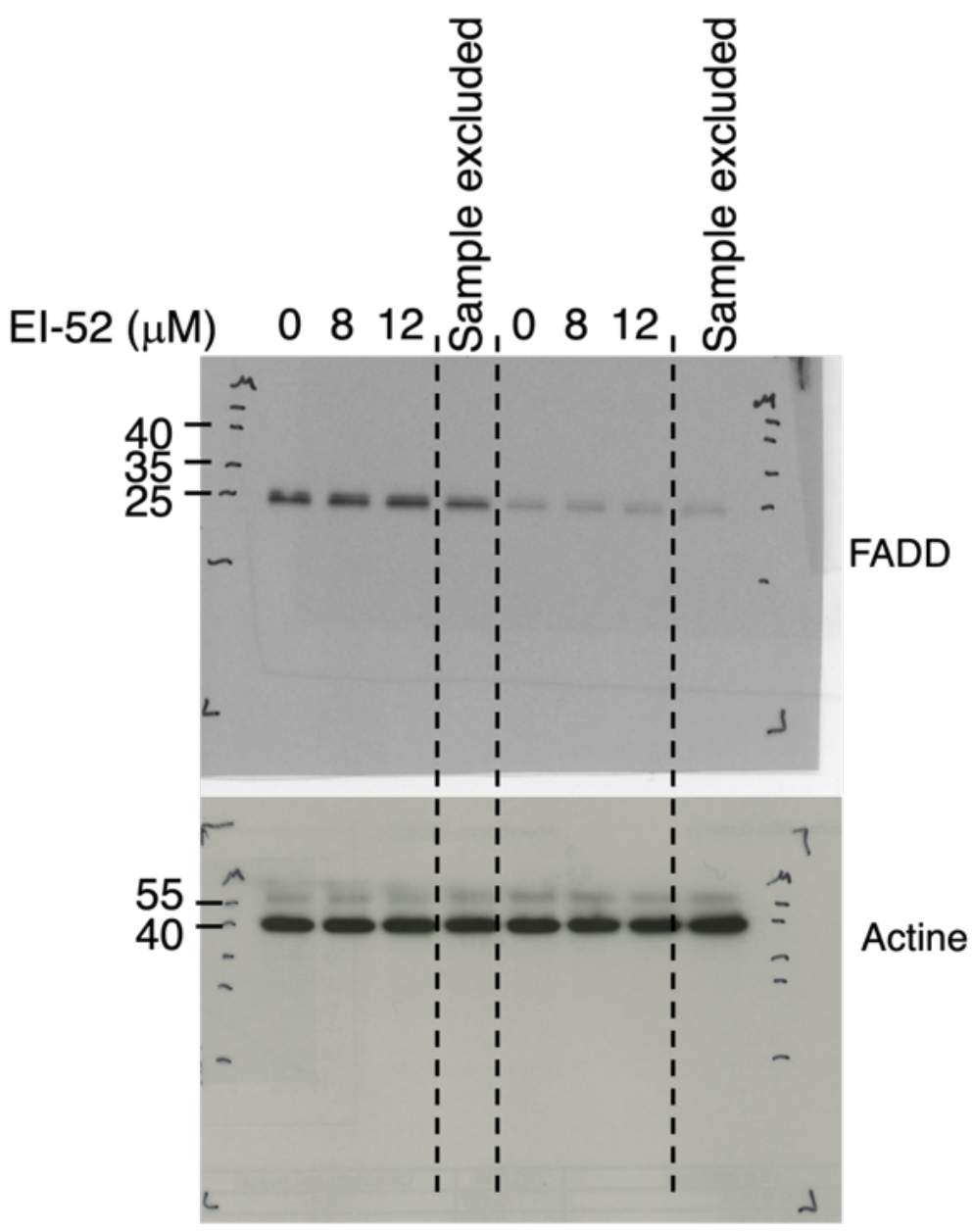

Supplementary Figure 1e

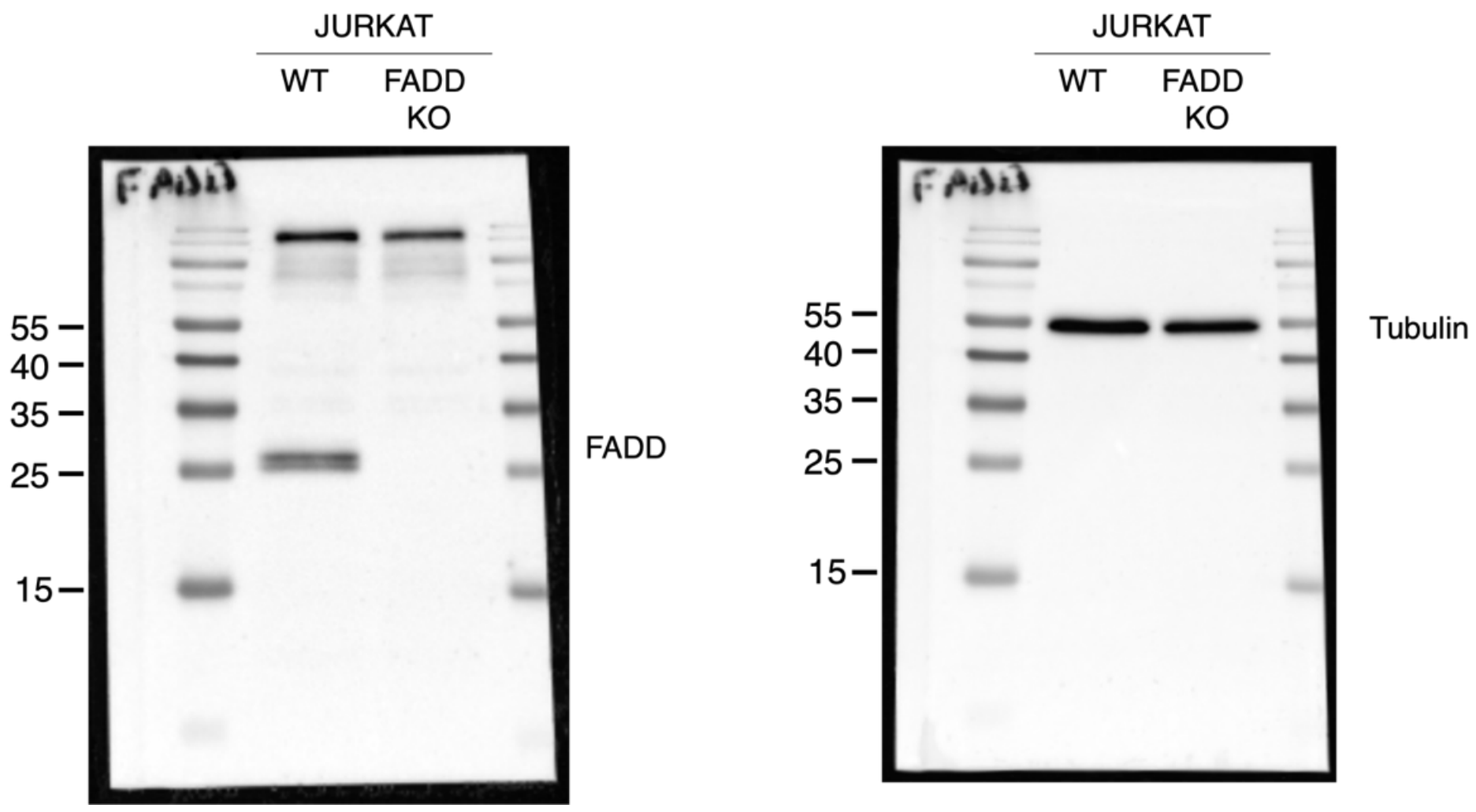

Supplementary Figure 1f

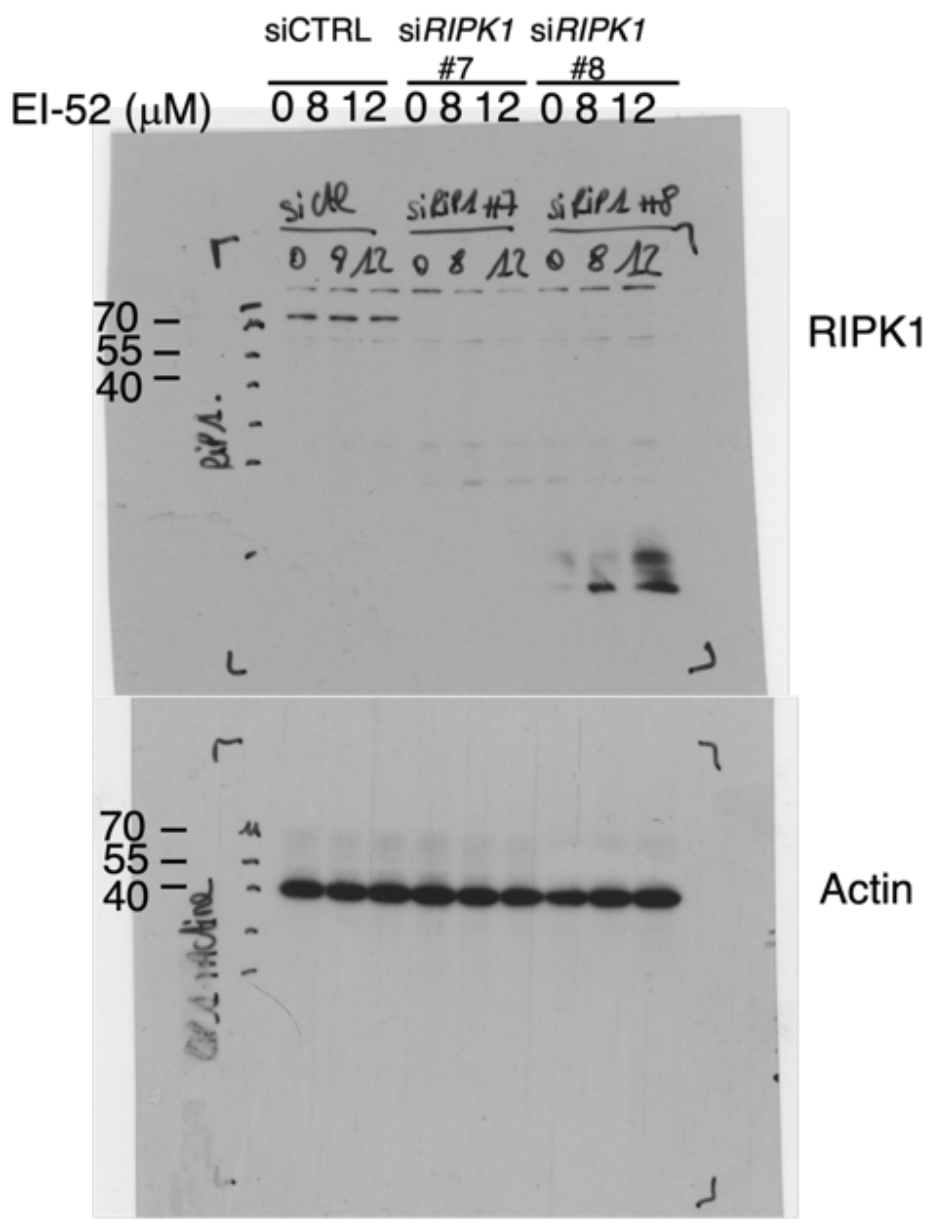

Supplementary Figure 2b

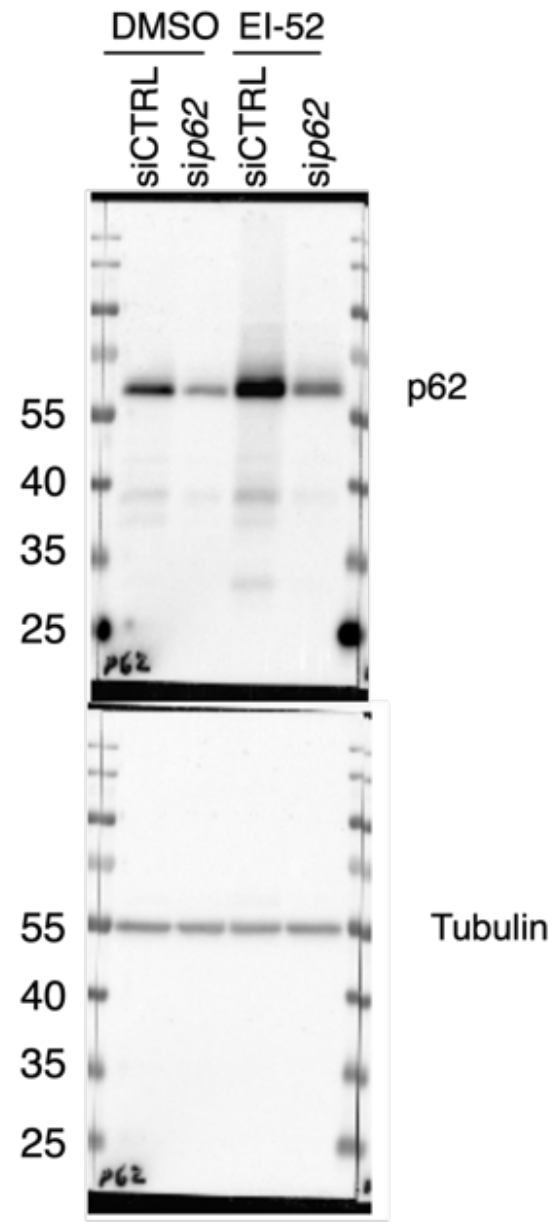

Supplementary Figure 3a

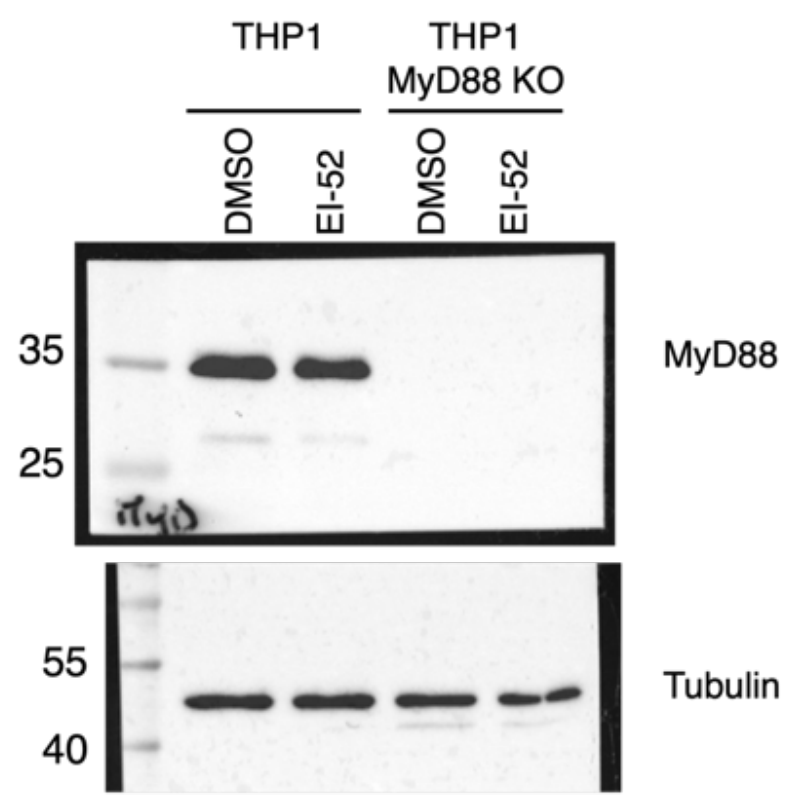

Supplementary Figure 3b

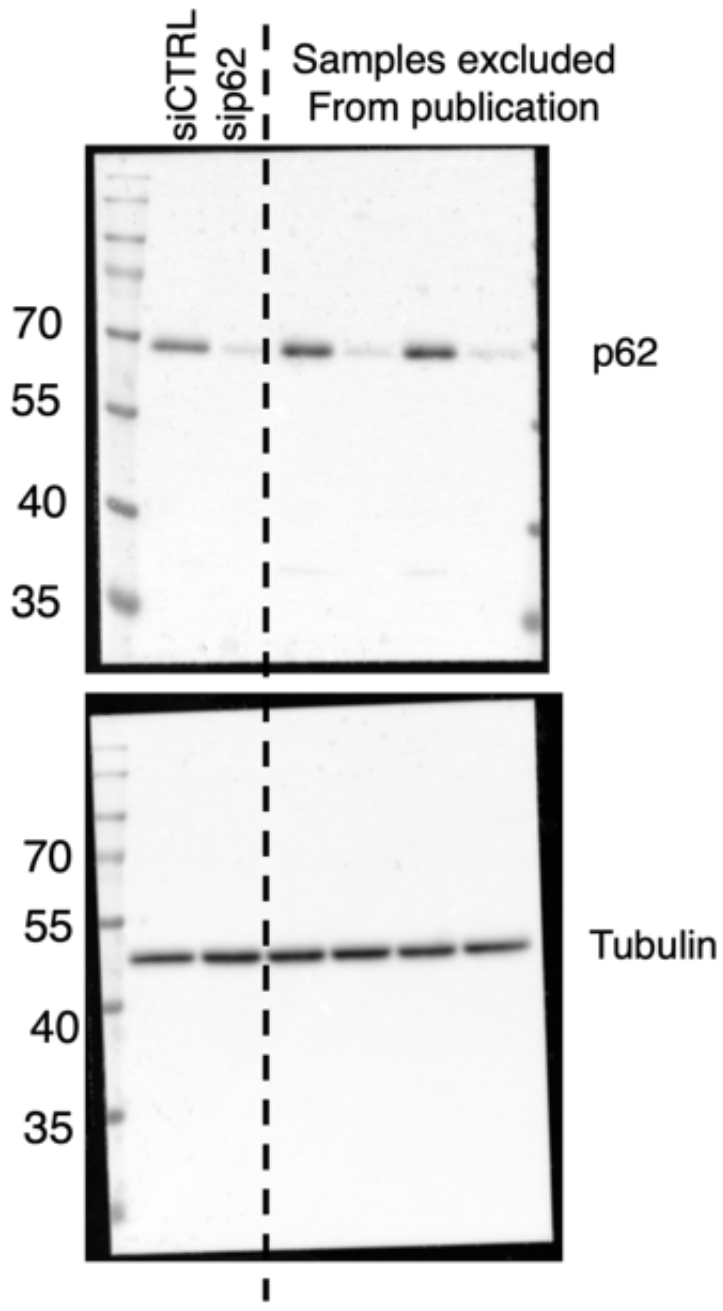

Supplement: Supplementary file 2 [file 41419_2026_9131_MOESM2_ESM.pdf]
